# Using Hybrid PDI-Fe_3_O_4_ Nanoparticles for Capturing Aliphatic Alcohols: Halogen Bonding vs. Lone Pair–π Interactions

**DOI:** 10.3390/ijms25126436

**Published:** 2024-06-11

**Authors:** María de las Nieves Piña, Alberto León, Antonio Frontera, Jeroni Morey, Antonio Bauzá

**Affiliations:** Department of Chemistry, Universitat de les Illes Balears, Ctra. de Valldemossa km. 7.5, 07122 Palma de Mallorca, Islas Baleares, Spain; neus.pinya@uib.es (M.d.l.N.P.); albertoperezleon@hotmail.com (A.L.); toni.frontera@uib.es (A.F.)

**Keywords:** Fe_3_O_4_ nanoparticles, perylene diimides, noncovalent interactions, thermal desorption experiments, DFT calculations, lone pair–π vs. halogen bond

## Abstract

In this study, Fe_3_O_4_ nanoparticles (FeNPs) decorated with halogenated perylene diimides (PDIs) have been used for capturing VOCs (volatile organic compounds) through noncovalent binding. Concretely, we have used tetrachlorinated/brominated PDIs as well as a nonhalogenated PDI as a reference system. On the other hand, methanol, ethanol, propanol, and butanol were used as VOCs. Experimental studies along with theoretical calculations (the BP86-D3/def2-TZVPP level of theory) pointed to two possible and likely competitive binding modes (lone pair–π through the π-acidic surface of the PDI and a halogen bond via the σ-holes at the Cl/Br atoms). More in detail, thermal desorption (TD) experiments showed an increase in the VOC retention capacity upon increasing the length of the alkyl chain, suggesting a preference for the interaction with the PDI aromatic surface. In addition, the tetrachlorinated derivative showed larger VOC retention times compared to the tetrabrominated analog. These results were complemented by several state-of-the-art computational tools, such as the electrostatic surface potential analysis, the Quantum Theory of Atoms in Molecules (QTAIM), as well as the noncovalent interaction plot (NCIplot) visual index, which were helpful to rationalize the role of each interaction in the VOC···PDI recognition phenomena.

## 1. Introduction

The perseverance of volatile organic compounds (VOCs) in the atmosphere constitutes an important environmental challenge, with deep implications for air quality, human health, and ecosystem integrity [1,2,3,4,5,6]. VOCs encompass a series of organic chemicals characterized by a high vapor pressure and a low boiling point at room temperature, facilitating their release into the atmosphere from various anthropogenic and natural sources [7,8,9,10].

In this context, aliphatic alcohols constitute a broad category of organic compounds ubiquitous in various sectors, including industrial processes [11,12], transportation [13,14], agriculture [15,16], and household activities [17,18]. Their environmental impact expands beyond their direct emissions, encompassing a variety of processes and interactions within the atmosphere. More in detail, these compounds undergo photochemical reactions with atmospheric oxidants, such as hydroxyl radicals and ozone, leading to secondary pollutants (e.g., formaldehyde, acetaldehyde, and peroxyacetyl nitrate) upon their release [19,20,21]. These secondary products not only further contribute to air quality degradation but are also involved in the formation of tropospheric ozone as well as the increase in smog and haze in urban and industrialized regions [22,23].

Aliphatic alcohols also serve as precursors for the formation of secondary organic aerosols through gas-to-particle conversion processes, thus further influencing atmospheric radiative forcing, cloud formation, and regional climate patterns [24,25]. In this context, the deposition of VOCs and their oxidation products onto terrestrial and aquatic ecosystems can exert adverse ecological effects, ranging from phytotoxicity and nutrient imbalances to the disruption of microbial communities and biogeochemical cycles [26,27,28]. On the other hand, their implications for human health span from respiratory disorders [29] to neurological impairments [30] and carcinogenic effects [31]. Moreover, alcohols are also involved in accidents and incidents occurring at fuel ethanol and biodiesel facilities around the world, where inadequate work or maintenance procedures have led to personal and material losses [32,33].

Therefore, the design and use of novel materials capable of efficiently capturing aliphatic alcohols is of paramount importance to improve human health and air quality as well as to mitigate their impact on ecosystems. In this regard, our group previously used hybrid magnetic nanoparticles (NPs) to capture and retain aromatic and aliphatic VOCs. This material proved to have great thermal stability, and it can be reused more than 200 times without losing its adsorption capacity [34,35]. Magnetic nanoparticles of iron oxide (Fe_3_O_4_) possess a series of advantages compared to other materials, such an easier preparation routine and functionalization as well as an efficient recovery process once used, owing to their sensitivity to external magnetic fields [36]. Hence, they have become an attractive alternative to conventional VOC-capturing materials from the perspective of reuse and environmental sustainability [37].

**Figure 1:**
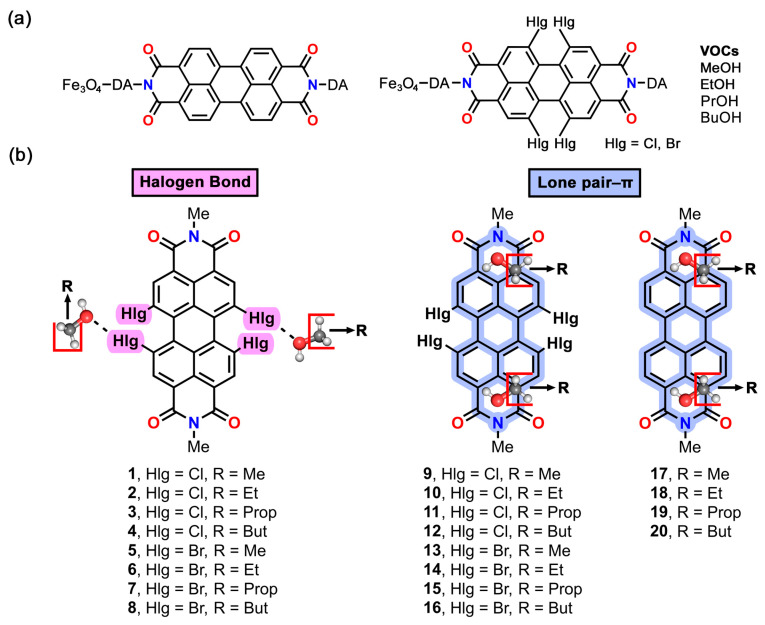
(**a**) PDIs and VOCs used in this study; (**b**) complexes **1** to **20** studied herein. A–Me group has been used as a replacement for the DA (dopamine) and Fe_3_O_4_ moieties at both sides of the PDI molecule.

Building upon these previous successes, in this study, hybrid Fe_3_O_4_ nanoparticles (FeNPs) decorated with different perylene diimides (PDIs) were used to capture and retain alkyl alcohols. Concretely, we have used tetrachlorinated and tetrabrominated PDIs (Cl-PDI and Br-PDI, respectively) as well as a nonhalogenated PDI to capture methanol, ethanol, propanol, and butanol. This has been performed by combining thermal desorption (TD) experiments with theoretical calculations at the BP86-D3/def2-TZVPP level of theory, which have shed light onto the noncovalent interactions (NCIs) involved in the VOC···PDI recognition phenomena. The TD experiments pointed to two main different recognition modes, which were confirmed by means of theoretical calculations, being based on halogen bond (HlgB) and lone pair–π (lp-π) interactions (see Figure 1 below). Additionally, the Quantum Theory of Atoms in Molecules (QTAIM) and the noncovalent interaction (NCIplot) visual index were used to further characterize the NCIs present in the supramolecular assemblies studied herein. We expect the results reported in this manuscript will be useful for those scientists working in the fields of supramolecular chemistry and materials science by providing new insights into novel recognition modes (e.g., HlgB) between aliphatic VOCs and FeNPs.

## 2. Results and Discussion

### 2.1. Synthesis and Characterization of the Nanoparticles, Fabrication of the Sorbent Tubes, and Adsorption/Desorption Experiments

The preparation of the hybrid nanoparticles was carried out by using the methodology developed by our research group. Briefly, magnetite nanoparticles were obtained by the coprecipitation method from Fe(II) and Fe(III) iron salts [38]. The PDI moiety was synthetized by adding two equivalents of dopamine hydrochloride to one equivalent of perylene-3,4,9,10-tetracarboxylic bisanhydride in water [37]. To obtain the Br-PDI moiety, firstly, the perylene-3,4,9,10-tetracarboxylic bisanhydride was brominated with Br_2_ in a sulfuric media; then, one equivalent of this 1,6,7,12-tetrabromoperylene-3,4,9,10-tetracarboxylic acid bisanhydride was combined with two equivalents of dopamine hydrochloride in a *N*,*N*-dimethylformamide (DMF)/water mixture [39]. The Cl-PDI moiety was obtained by the combination of 1,6,7,12-tetrachloroperylene-3,4,9,10-tetracarboxylic acid bisanhydride (commercial product) with two equivalents of dopamine hydrochloride in a DMF/water mixture (see the Section 3 for experimental details and Supplementary Materials for characterization). Subsequently, the conjugation of the PDI, Br-PDI, and Cl-PDI moieties with the magnetite was carried out by using the microwave-assisted heating method, as it significantly increases the covalent functionalization of the nanoparticles [40].

The FT-IR spectrum (Figure S3), the TEM microphotograph (Figure S4), and the thermogravimetric analysis (Figure S5) corresponding to the Cl-PDI-NP are shown in the Supplementary Materials. In the IR spectrum (Figure S3), it can be observed that the corresponding bands are related to the magnetite and dopamine-Cl-PDI, indicating the presence of both in the final product. By means of the TGA (Figure S5), we demonstrated that the NP and the dopamine-Cl-PDI fragment are connected to each other, which is revealed by the loss of weight located between 200 and 800 °C. The percentage that appears in the TGA analysis corresponds to the loss of weight due to the volatilization of the PDI-NP coating, being at 43%. This corresponds to the NP surface coating percentage. On the other hand, TEM microphotography (Figure S4) allowed us to check that the nanoparticles are of the same size (between 50 and 70 nm) and present a spherical shape.

The sorbent tubes were prepared by using TD glass tubes of 6 mm in diameter and 90 mm in length, which were filled with 250 mg of hybrid nanoparticles, and unsilanized glass wool was put at both ends. Thus, nine TD tubes were prepared, three with PDI-NPs, three with Br-PDI-NPs, and three with Cl-PDI-NPs.

The methodology used to determine the capability of the sorbent tubes is the same as described in previous experiments [34]. Initially, we used one tube for the generation of the calibration plots (see Supplementary Materials Figures S6–S17). and then two tubes linked by a Swagelock adapter were used to determine the adsorption percentages. For the doping of the tubes, a commercial doping device that allows for the manual injection of the VOC through a Swagelock adapter was used. This adapter connects the tube without any possibility of leakage. The N_2_ flow that constantly goes through the device ensures the homogeneity of the sample across the sorbent. For the sake of reproducibility, we always used 1 μL of VOC solution and a doping time of 5 min (see the Section 3 for details). The standard solutions were freshly prepared. The concentration range of the VOCs was between 0.11 and 0.55 mg (see Supplementary Materials Figure S18). Retention tests were carried out with a solution containing 0.33 mg of analyte (see Supplementary Materials Figure S19). All of the tubes were periodically checked to test that they were able to give the same result throughout the entire process. After thermal cleaning, we were able to reuse the tubes >100 times without any loss of their adsorption properties.

### 2.2. Adsorption Results

As has been demonstrated in previous studies [34], although non-functionalized magnetite nanoparticles show a certain retention capacity for non-aromatic VOCs, after a few days and several cycles, they suffer leaching processes that considerably reduce their adsorption capacity. On the other hand, PDI moieties themselves are not suitable to act as a collector, since their powdery nature makes impossible their use for that purpose. This fact demonstrates the effectiveness of the combination of nanoparticles with PDI moieties, since the new materials have great thermal stability and ease of handling. The results for each sorbent and VOC pair are gathered in Table 1. The values presented have been obtained as the average of three independent experiments for each pair. Although MeOH does not follow the same trend as the rest of the VOCs, it is clearly observed that the retention capacity of the NPs increases with the addition of halogens in the PDI structure. Of the two halogens tested, in all cases, the Br-PDI-NP presents higher retention percentages than the Cl-PDI-NP. On the other hand, if the values obtained for the four alcohols are compared, only in the case of Br did we observe that, as the chain size increases, the retention percentage also increases, likely due to a larger surface of interaction between the alcohol molecule and the PDI surface. This is not observed for the PDI and Cl-PDI systems, where the MeOH exhibits the largest retention percentages, which could possibly be due to a different VOC-PDI recognition mechanism compared to the rest of alcohols or a different VOC-PDI stoichiometry compared to the rest of alcohols, which follow the same trend as the Br-PDI system.

### 2.3. Theoretical Calculations

Since the PDI acts as a VOC recognition unit, we have focused the computational part of this manuscript on the PDIs’ electronic structure and their ability to interact with the alcohol moieties. More in detail, we have started by analyzing the electrostatic potential surfaces of the Cl-PDI, Br-PDI, and PDI molecules, followed by calculations regarding two plausible VOC-PDI recognition modes based on the HlgB and lp-π interactions, respectively. Lastly, the physical nature of the noncovalent complexes studied herein has been studied from a charge-density perspective using the QTAIM and NCIplot methodologies.

### 2.4. Molecular Electrostatic Potential Surface Study

**Figure 2:**
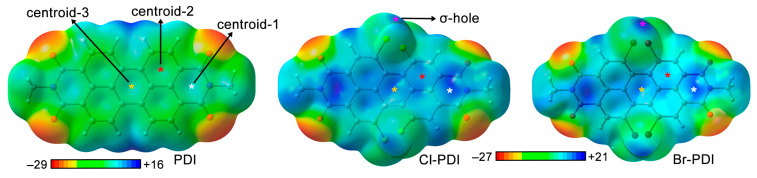
MEP surfaces of the tetrachlorinated (Cl-PDI), tetrabrominated (Br-PDI), and nonhalogenated PDI (PDI). Energy values at concreted points (denoted by white, red, orange, and purple stars) in the surface are given in kcal/mol (0.001 a.u.).

Owing to the π-acidic properties of the PDI moiety and the presence of halogen atoms, we conducted a Molecular Electrostatic Potential (MEP) surface analysis of the non-halogenated and halogenated PDIs (see Figure 2 and Table 2). As noticed, for both halogenated PDIs, similar electrostatic potential values were found over the PDI aromatic core (V_centroid-1/2/3_ in Table 1), ranging between +14 and +20.5 kcal/mol. These values are more positive than those corresponding to the nonhalogenated derivative, which were encompassed between +9.3 and +15.5 kcal/mol, thus expecting more favorable lp-π energies in those complexes involving halogenated PDIs from the point of view of electrostatics. If both halogenated PDIs are compared, the electrostatic term is not enough to predict which one will stablish the stronger lp-π interactions with the alcohol moieties, owing to their very similar values, thus pointing to other energy components (e.g., dispersion or polarization) as crucial to rationalize the trends observed in the interaction energies (see below).

On the other hand, the brominated PDI (Br-PDI) exhibited a more positive σ-hole MEP value (+19.8 kcal/mol) compared to the chlorinated one (Cl-PDI, +16.1), thus anticipating a stronger halogen bond binding with the electron rich O atom from the alcohol moiety in those complexes involving the former, as it is commonly observed [41]. Lastly, by comparing the halogen σ-hole MEP value with that corresponding to centroid-1 (which presents the largest π-acidity of all three rings), it can be anticipated that the lp-π binding mode will predominate and direct the molecular recognition phenomena between the PDI and the alcohol molecules.

### 2.5. Energetic Study

With the MEP results in mind, we computed the interaction energies of complexes **1** to **20** (see Table 3) involving the three PDI moieties as Lewis acids and MeOH, EtOH, PrOH, and BuOH as electron donor species. In all cases, the complexes were calculated using a 1:2 ratio between the PDI and the alcohol molecules. As noted, both halogenated PDIs exhibited larger interaction energy values than those complexes involving the nonhalogenated PDI, which was in line with the results from the MEP analysis discussed above and from the TD experiments. In addition, we also observed an increase in the interaction energy strength upon increasing the size of the alcohol molecule, which was also in agreement with the experimental results. Lastly, among the two halogenated PDIs, the interaction energies involving the lp-π binding mode (complexes **9** to **16**) were more favorable than those related to the HlgB complexes (**1** to **8**), which was in line with the MEP results discussed above.

**Figure 3:**
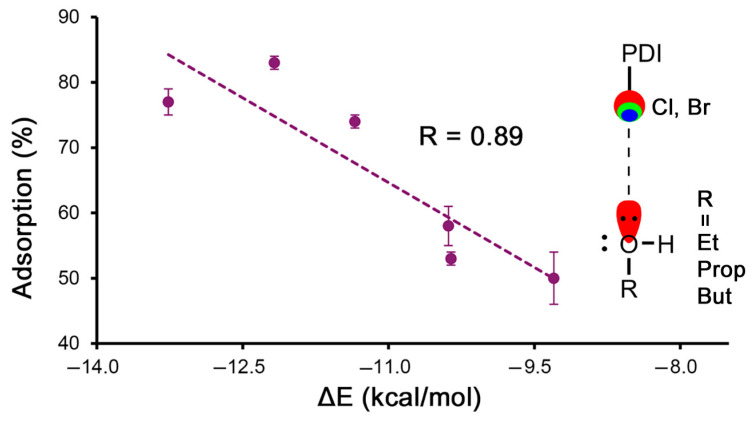
Regression plot of the % quantity of the VOC adsorbed and the interaction energies for the HlgB complexes **2** to **4** and **6** to **8**.

For the HlgB complexes **1** to **8**, the interaction energies spanned from −13.3 to −8.1 kcal/mol, being those complexes involving PrOH (**3** and **7**) and BuOH (**4** and **8**), the ones exhibiting the larger interaction energy values (e.g., complex **3** (PrOH@Cl-PDI), −10.4 kcal/mol, and complex **8** (BuOH@Br-PDI), −12.2 kcal/mol). While a clear increase in the interaction energy is observed from MeOH to EtOH and from EtOH to PrOH, complexes involving PrOH and BuOH achieved similar values in the case of the Cl-PDI (−10.4 kcal/mol for complexes **3** and **4**), while complex **7** involving PrOH and Br-PDI achieved a larger interaction energy value (−13.3 kcal/mol) than complex **8** involving BuOH (−12.2 kcal/mol). This difference of around 1 kcal/mol could be attributed to the different disposition of the CH groups from the aliphatic chain over the PDI, which affect the formation of CH-π interactions [42] with the PDI aromatic core (see Figure 3 below). Additionally, by comparing HlgB complexes **1** to **4** involving Cl-PDI with complexes **5** to **8** involving Br-PDI, we observed larger interaction energy values for those involving the latter, which was in line with the MEP analysis previously discussed.

For the lp-π complexes **9** to **16**, the interaction energy values ranged between −22.8 and −15.0 kcal/mol, with complexes **12** and **16** involving larger alcohols (PrOH and BuOH) being the most favorable ones, while complexes **9** and **13** involving MeOH were the least favorable ones of the set, which was in line with the results obtained for complexes **1** to **8**. Again, this might be due to the formation of ancillary i) CH-π interactions with the PDI’s π-system and ii) HC···CH interactions between the two alcohol moieties. In general, those complexes involving the Br-PDI achieved a larger interaction energy value than their corresponding Cl-PDI analogs, with the exception of complexes **12** and **16**, which showed a very similar strength (−23.0 and −22.8 kcal/mol), and also in a parallel way to the results obtained for the HlgB set. Lastly, we computed the interaction strength of the lp-π interaction using the nonhalogenated PDI (complexes **17** to **20**), achieving lower interaction energy values compared to the Cl-PDI and Br-PDI series (spanning between −21.3 and −7.2 kcal/mol), likely due to a decrease in the π-acidity of the PDI aromatic core (see Table 1).

Lastly, in Figure 3, we represented the percentage of adsorption vs. the interaction energies for those complexes involving the HlgB binding mode (**1** to **8**), with the exclusion of complexes **1** and **5** involving MeOH, owing to the different tendency observed in the results shown in Table 1. Remarkably, a good correlation between the adsorption percentages and the interaction energies was found (R = 0.89). Such a correlation supports the HlgB-based recognition mode between the VOCs and the Cl/Br atoms from the PDI moiety while also representing a direct correlation between the computational interaction energies of the HlgB complexes and the experimental adsorption data. On the contrary, if the same representation is carried out in the case of the lp-π-based complexes (**9** to **20**), again, without considering complexes **9**, **13**, and **17** involving MeOH, a poor correlation coefficient was obtained between the adsorption percentages and the interaction energies (R = 0.60), indicating that the VOC recognition mechanism over the PDI π-surface is not solely based on a combination of lp-π and CH-π interactions.

**Figure 4:**
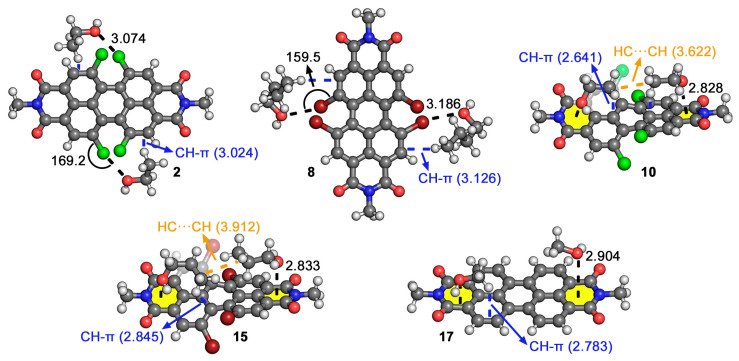
Optimized geometries of complexes **2**, **8**, **10**, **15**, and **17** at the BP86-D3/def2-TZVPP level of theory. Ancillary CH-π and CH···HC interactions are highlighted in blue and orange, respectively. The intermolecular distances and angles are also indicated in Å and °, respectively.

The geometries of some of the optimized complexes are shown in 0 in the case of the HlgB complexes **2** and **8**, the alcohol molecules surround the Cl and Br of the Hlg-PDI, establishing two simultaneous HlgBs with a high directionality (C–Cl···O and C–Br···O angles of 169.2 and 159.5°, respectively). In addition, the alkyl chains are also relatively close to the Hlg-PDI core, establishing CH-π interactions with a PDI aromatic system (denoted in blue in Figure 4). On the other hand, in the case of the lp-π complexes **10**, **15**, and **17**, two alcohol molecules interact with the PDI aromatic core by means of lp-π interactions, involving the six members that presented the largest π-acidity of all three (the most positive MEP value in Table 1). In addition, CH-π interactions were undertaken between the CH groups from the alcohol alkyl chain and the aromatic π-system of the PDI molecule and CH···HC interactions between the two alcohol molecules (highlighted in orange in Figure 4), which also contributed to the stabilization of the VOC particles over the PDI surface.

### 2.6. QTAIM and NCIplot Analyses

**Figure 5:**
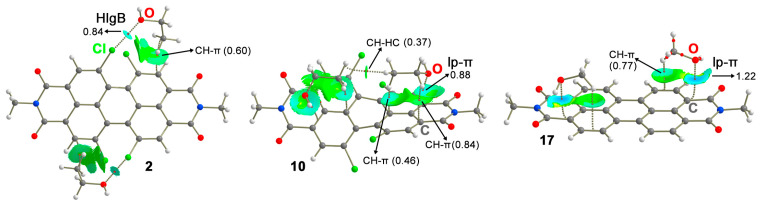
Distribution of intermolecular bcps (red dots) and bond paths in complexes **2**, **10**, and **17**. Ancillary CH···HC and CH-π interactions are also indicated. The values of the density (ρ·10^2^) related to the HlgB, lp-π, as well as ancillary interactions are also included in a.u. NCIplot color range −0.02 au ≤ (signλ_2_)ρ ≤ +0.02 au.

With the purpose of analyzing the HlgB and lp-π complexes from a charge-density perspective, we computed the QTAIM analyses of several representative complexes (see Figure 5). As noticed, in the case of the HlgB complex **2**, the HlgB is described by the presence of a bond critical point (bcp) and bond path connecting the Cl atoms from the Cl-PDI to the O atoms from the alcohol molecules. In addition, ancillary CH-π interactions are characterized by bcps that connected the CH groups from the aliphatic chain belonging to the alcohol moieties to the C atoms from the Cl-PDI aromatic system.

In the case of lp-π complexes **10** and **17**, the interaction is denoted by a bcp that connects the O atom from the alcohol moieties to the C atoms from PDI molecule core. Furthermore, the ancillary CH···HC and CH-π interactions are also denoted by the presence of several bcps and bond paths that connected the CH groups from the alcohol alkyl chain to (i) aliphatic CH groups from the vicinal alcohol molecule and (ii) to the π-system of the Cl-PDI. Lastly, in both types of complexes, the value of the density at the bcp that describes either the HlgB or the lp-π interaction is of a larger magnitude than that observed for the ancillary CH···HC and CH-π interactions, denoting a predominant role of the HlgB and lp-π bonds in the formation of the supramolecular assemblies studied herein.

Lastly, we have also computed the NCIplot analysis of these three systems, showing green and blue isosurfaces between both counterparts that further assisted on the identification and characterization of the NCIs present in these supramolecular assemblies. For both types of complexes, the NCI isosurface corresponding to the HlgB and lp-π interaction presents a more bluish color compared to that observed for the HC···HC and CH-π interactions, thus confirming the directing role of the former interactions in the alcohol···PDI molecular recognition phenomena, in agreement with the QTAIM analyses discussed above.

## 3. Materials and Methods

### 3.1. Experimental Methods

#### 3.1.1. General

Reactions were carried out in oven-dried glassware under an atmosphere of argon, unless otherwise indicated. High-purity water was generated by the Milli-Q apparatus (Millipore). The synthesis and characterization of the nanoparticles decorated with PDI and Br-PDI moieties used in this work have been previously described by us [37,38,39]. All of the commercially available reagents, including dopamine hydrochloride, triethylamine, perylene 3,4,9,10-tetracarboxylic bisanhydride, and 1,6,7,12-tetrachloroperylene-3,4,9,10-tetracarboxylic acid bisanhydride, and the VOC analytes (purity higher than 98%) were purchased from Sigma-Aldrich (St. Louis, MO, USA) and Fluka (Merk Life Science S.L.U., Madrid, Spain). All of the solvents were purchased from Scharlab (Sharlab S.L., Barcelona, Spain). The glass tubes (6 mm in diameter and 90 mm in length) and the unsilanized glass wool were purchased from Supelco (Bellefonte, PA, USA). All of the nanoparticles are thermally stable up to 400 °C, therefore they are suitable for utilization in the experiments detailed below, as the maximum temperature used in the thermal desorption is 375 °C. No degradation of the magnetic nanoparticles was detected even after 100 repetitions of the experiment.

#### 3.1.2. Instrumentation

The ^1^H and ^13^C NMR spectra were recorded on a Bruker Advance Spectrometer (Bruker Española S.A., Madrid, Spain) at 300 and 75 MHz at 25 °C. Chemical shifts were reported as a part per million (δ, ppm) referenced to the residual protium signal of the deuterated solvents. Spectral features were tabulated in the following order: chemical shift (δ, ppm); multiplicity (s—singlet, d—doublet, t—triplet, and m—multiplet); the number of protons (FTIR) were obtained on a Bruker Tensor 27 instrument (Bruker Española S.A., Madrid, Spain) in solid-state. Matrix-assisted laser desorption/ionization mass spectra (MALDI) were recorded with an Autoflex III MALDI TOF/TOF mass spectrometer provided with a Smartbeam Laser at 200 Hz (Bruker Española S.A., Madrid, Spain). Functionalization of the iron nanoparticles was performed on a Biotage Initiator Classic Microwave Synthesizer (Biotage, NASDAQ, Stockholm) at 400 W and 2 bar. Thermal analysis was recorded on a TA Instruments model SDT 2960 (TA Instruments, New Castle, DE, USA). Scanning Electron Microscopy (SEM) microphotography was obtained on a HITACHI S-3400N (HITACHI in Spain, Madrid, Spain).

#### 3.1.3. Preparation of 2,9-Bis(3,4-Dihydroxyphenethyl)-1,6,7,12-Tetrachloroperylene Tetracarboxylic Diimide (Cl-PDI)

In a round-bottom flask was dissolved 0.158 g (0.250 mmol) of 1,6,7,12-tetrachloroperylene-3,4,9,10-tetracarboxylic acid bisanhydride in 20 mL of a 1:1 (*v*/*v*) mixture of H_2_O and DMF. Then, 2 mL of Et_3_N were added and stirred for more than 2 h. After that, 99 mg (0.522 mmol) of dopamine hydrochloride was dissolved in 10 mL of a 1:1 (*v*/*v*) mixture of H_2_O and DMF, and was added dropwise to the first flask. Once the addition was complete, the mixture was refluxed overnight. The oil obtained was allowed to temper and was transferred to a falcon tube, where concentrated HCl was added until a brown-reddish precipitate was formed. The crude was centrifuged and washed repeatedly with Milli-Q water until the neutrality of the supernatant liquid was observed. The product was dried in a vacuum at 120 °C for 8 h and obtained as a reddish-brown powder at a yield of 115 mg (0.144 mmol, 58% yield). ^1^H NMR (300 MHz, D_2_O-NaOD): ∂: 8.04 (d, 4H), 6.86 (m, 4H), 6.77 (m, 2H), 3.25 (t, 4H), and 2.62 (t, 4H) (see Supplementary Materials Figure S1). ^13^C NMR (300 MHz, D_2_O-NaOD): ∂: 176.4 (C=O), 137.8 (C=C), 131.3 (C-Cl), 128.3 (C=C), 127.9 (C=C), 126.7 (C=C), 116.1 (C=C), 45.3 (CH_2_), and 39.5 (CH_2_) (see Supplementary Materials Figure S2). FTIR (KBr): ν = 3439, 1716, 1633, 1591, 1557, 1431, 1338, 1361, 810, and 768 cm^−1^_._ MALDI-TOF-MS *m*/*z* (%): [M]^+^ calculated for C_40_H_22_Cl_4_N_2_O_8_ 798.0130 found 798.0125.

#### 3.1.4. Preparation of Functionalized Magnetite Nanoparticles Cl-PDI-NP

In a microwave tube was introduced 25.26 mg (0.026 mmol) of 2,9-bis(3,4-dihydroxyphenethyl)-1,6,7,12-tetrachloroperylene tetracarboxylic diimide with 4 mL of Milli-Q water, one drop of 1M NaOH, and 1 mL of magnetite nanoparticle suspension (11.4 mg/mL). The mixture was sonicated for 5 min and then introduced in the microwave reactor. The reaction conditions of the microwave reactor were as follows: 120 °C, 3 bar, and 30 min of reaction time. Once the reaction was over, the nanoparticles were decanted with the help of a boron-neodymium magnet and washed 3 times with EtOH. Finally, the hybrid nanomaterial was suspended and stored in 10 mL of EtOH in an argon atmosphere. FTIR (KBr): *ν* = 3442, 2752, 2540, 1637, 1592, 1434, 852, and 598 cm^−1^.

#### 3.1.5. Standard Solutions

Stock standard solutions for each VOC were freshly prepared in acetone for each experiment and stored at 4 °C in the dark in 1.0 mL flasks. These mother solutions were further diluted in acetone to prepare standard solutions of 0.11, 0.22, 0.33, 0.44, and 0.55 mg, with a final volume of 500 μL. Before starting the experiments, it was verified that the acetone did not show significant retention by any of the absorbents so that it would not interfere with the results obtained.

#### 3.1.6. Tubes Filled with Hybrid Nanoparticles

One tube for the generation of the calibration plots and two tubes connected in a series were used to determine the percentage of the retention of the analyte in the functionalized nanoparticles. Each tube was filled with 250 mg of hybrid nanoparticles and, periodically, it was checked that both tubes were able to give the same results when doped with the same quantity of a given VOC. After total desorption, both tubes were checked to make sure that they were perfectly clean. Before the first use, the tubes were conditioned by thermal cleaning (250 °C for 60 min, 300 °C for 60 min, 350 °C for 60 min, and 375 °C for 60 min) under a flow rate of dry N_2_ of 100 mL min^−1^. For subsequent uses, pre-conditioning at 375 °C for 60 min was applied. After conditioning, they were immediately sealed with Swagelock end-caps fitted with PTFE ferrules and stored in closed plastic boxes filled with desiccant material. 

For the doping of the tubes, the commercial doping device supplied by the instrument manufacturer was used. An amount of 1 μL of the VOC solution was used with a doping time of 5 min and, during the first minute, the syringe was kept in the injector to maintain the flux unperturbed.

All of the materials presented in this study are stable in air and keep their properties unchanged. The use of dry N_2_ flow is necessary for the correct operation of the doping device and thermal desorption. Sealing the tubes with Swagelock caps after conditioning is simply to ensure that they do not become contaminated with products present in the laboratory environment.

#### 3.1.7. Analytical Instrumentation and Procedure

The VOC analyses were carried out by using an autosampler (Ultra-xr) coupled to thermal desorption (TD) (Unity 2 model, Markes International, Bridgend, UK) and coupled to a gas chromatograph with an FID detector (6890A model, Agilent Technologies, Santa Clara, CA, USA). The first step was an initial pre-desorption for 0.1 min and a flux of 20 mL min^−1^. Subsequently, a primary desorption was performed at 300 °C for 10 min, with a split of 4 mL min^−1^. During this period, the analyte that was previously adsorbed in the tube was concentrated in a cryofocusing trap that contained 30 mg of Tenax TA and which was kept at −30 °C. Afterwards, the trap was quickly heated from −30 °C to 300 °C and kept at the final temperature for 10 min and, thus, the secondary desorption took place. In this step, the analyte was sent to the gas chromatograph by using a split with a flux of 7 mL min^−1^ and finally injected into a capillary column (DB-624, 60 m × 0.25 mm × 1.4 mm, Agilent Technologies) through a line at 200 °C. The oven temperature was initially set at 40 °C for 1 min and progressively increased (rate of 6 °C min^−1^) until 230 °C. This final temperature was maintained for 5 min. The carrier gas was premium quality N_2_ with a flux of approximately 1 mL min^−1^.

### 3.2. Computational Methods

Theoretical calculations were performed at the BP86-D3 [43,44]/def2-TZVPP [45] level of theory by means of the TURBOMOLE 7.7 program [46]. During the theoretical modeling process, the Fe_3_O_4_ and dopamine moieties were replaced by—Me groups at both sides of the PDI molecule, which acted as the VOC recognition unit, as demonstrated in previous studies [34,35]. The interaction energies gathered in Table 1 were calculated using the supermolecule approximation (ΔE_NCI_ = E_PDI_ − 2 × E_ROH_). In the case of complexes **9** to **20**, the contribution of the CH-HC interactions to the total interaction energy was subtracted by performing a single-point calculation of the two alcohol moieties using their disposition in the complex. The energies corresponding to this set of complexes only contain the contribution of lp-π and CH-π interactions between the alcohol molecules and the PDI π-system.

The MEP surfaces were computed at the same level of theory using the Gaussian 16 calculation package [47] and visualized using the Gaussview 5.0 program [48]. In addition, the wavefunction analyses were performed using the AIMall software (v19.10.12) [49]. Lastly, the NCIplot isosurfaces (v19.10.12) [50] corresponded to both favorable and unfavorable interactions, as differentiated by the sign of the second-density Hessian eigenvalue and defined by the isosurface color. The color scheme was a red-yellow-green-blue scale, with red for a repulsive (ρ_cut_^+^) and blue for an attractive (ρ_cut_^−^) NCI interaction density. Yellow and green surfaces corresponded to weak repulsive and weak attractive interactions, respectively. The surfaces were visualized using the Visual Molecular Dynamics (VMD) software (v1.9.3) [51].

## 4. Conclusions

In this study, we used FeNPs functionalized with halogenated PDIs to capture aliphatic alcohols in addition to a nonhalogenated PDI that was used as a reference system. The newly prepared Cl-PDI derivative was synthesized and obtained with good performance and optimal functionalization. These FeNPs functionalized with halogenated PDIs demonstrated remarkable thermal stability, and no loss of retention capacity was observed after 100 adsorption cycles. Comparative adsorption/desorption experiments demonstrated that the presence of halogens in the structure of the PDI moiety considerably increased the interaction capacity between the adsorbent and the aliphatic VOCs tested. Specifically, the bromoderivative material exhibited the highest retention percentages. Regarding the size of the VOC, it is observed that retention increases as the chain lengthens.

On the other hand, computations at the BP86-D3/def2-TZVPP level of theory agreed with these experimental results and revealed two plausible noncovalent binding modes between the alcohol molecules and the PDIs, involving HlgB and lp-π bonds. The results showed that those HlgB and lp-π complexes involving Br-PDI exhibited more favorable interaction energy values, which was in line with the results derived from the MEP study and TD experiments, which revealed higher retention percentages when using Br-PDI. In addition, a reinforcement of the strength of the NCIs was also observed while increasing the alcohol chain length. Additionally, between both types of binding modes, the lp-π complexes resulted in larger and more favorable interaction energies compared to their HlgB analogs, which was also due to the formation of CH-π interactions between the alcohol aliphatic chain and the PDI aromatic core, as well as the HC···CH interactions between the alcohol alkyl chains, which certainly contributed to the stabilization of the supramolecular assemblies. Moreover, when using the nonhalogenated PDI, lower interaction energy values were obtained compared to the Hlg-PDIs, which was also in line with the electrostatic potential surface analysis and the experimental results, which showed the almost lower VOC retention percentages when using the nonhalogenated PDI. Lastly, the HlgB and lp-π complexes analyzed herein were also studied by means of the QTAIM and NCIplot methodologies, which confirmed the weak and attractive nature of the interactions and the director role of either the HlgB or lp-π bonds in the formation of the complexes studied herein.

We expect the results reported herein will be useful for those scientists working in the design of adsorbents specifically designed for medium-chain aliphatic VOCs, which are often difficult to retain by conventional materials, as well as to supramolecular chemists by providing new insights into the VOC recognition mechanism of halogenated PDIs.

## Figures and Tables

**Table 1:** Retention time of the VOC in minutes (RT), milligrams of the VOC in the front tube, and the absorption as a percentage of the VOC retained in the back tube for each material/VOC combination. Data collected from three independent experiments performed in different sessions.

Material	VOC	RT (min)	VOC in Front Tube (mg)	Adsorption (%)
PDI-NP	MeOH	6.98	0.139 ± 0.019	58 ± 5
Cl-PDI-NP	MeOH	6.98	0.106 ± 0.016	68 ± 5
Br-PDI-NP	MeOH	6.98	0.093 ± 0.016	72 ± 5
PDI-NP	EtOH	8.33	0.248 ± 0.009	25 ± 3
Cl-PDI-NP	EtOH	8.33	0.165 ± 0.013	50 ± 4
Br-PDI-NP	EtOH	8.33	0.086 ± 0.003	74 ± 1
PDI-NP	PrOH	11.3	0.232 ± 0.009	29 ± 3
Cl-PDI-NP	PrOH	11.3	0.155 ± 0.007	53 ± 1
Br-PDI-NP	PrOH	11.3	0.076 ± 0.007	77 ± 2
PDI-NP	BuOH	14.8	0.227 ± 0.008	31 ± 3
Cl-PDI-NP	BuOH	14.8	0.139 ± 0.011	58 ± 3
Br-PDI-NP	BuOH	14.8	0.056 ± 0.004	83 ± 1

**Table 2:** Electrostatic potentials (V, in kcal/mol) computed at the Cl/Br σ-hole (V_σ-hole_) as well as several parts of the PDI aromatic core (V_centroid-1_, V_centroid-2_, and V_centroid-3_, see Figure 2). The MEP minima (V_min_) and maxima (V_max_) are also indicated.

Material	V_σ-hole_	V_centroid-1_ *	V_centroid-2_ *	V_centroid-3_ *	V_min_	V_max_
Cl-PDI	+16.1	+19.9	+17.3	+14.1	−27	+21
Br-PDI	+19.8	+20.5	+16.4	+14.0	−27	+21
PDI	-	+15.5	+12.8	+9.3	−29	+16

* Values gathered at the center of each ring belonging to the PDI moiety (see Figure 2).

**Table 3:** Interaction energies (ΔE, in kcal/mol) of complexes **1** to **20** at the BP86-D3/def2-TZVPP level of theory. The intermolecular distances and angles (d and α, in Å and °, respectively) corresponding to the HlgB, lp-π, and ancillary interactions, as well as the values of the density at the bond critical point that characterizes the HlgB and lp-π and ancillary interactions (ρ × 100, in a.u.), are also indicated.

Complex (Material@VOC)	ΔE	ΔE per HlgB	d *	d *_CH-π (HC···CH)_	α	ρ × 100	ρ × 100 _CH-π (CH···HC)_
**1** (Cl-PDI@MeOH)	−8.1	−4.1	3.053	3.074	160.3	0.99	0.49
**2** (Cl-PDI@EtOH)	−9.3	−4.7	3.074	3.024	169.2	0.84	0.60
**3** (Cl-PDI@PrOH)	−10.4	−5.2	2.976	3.138	171.3	1.08	0.54
**4** (Cl-PDI@BuOH)	−10.4	−5.2	3.290	2.806	146.6	0.58	0.60
**5** (Br-PDI@MeOH)	−8.4	−4.2	3.054	2.849	159.9	1.07	0.58
**6** (Br-PDI@EtOH)	−11.3	−5.7	3.073	3.048	167.5	1.04	0.59
**7** (Br-PDI@PrOH)	−13.3	−6.7	2.953	3.183	174.2	1.38	0.52
**8** (Br-PDI@BuOH)	−12.2	−6.1	3.186	3.126	159.5	0.85	0.34
Complex	ΔE *	ΔE per lp-π	d *	d *_CH-π (HC···CH)_	ρ × 100		ρ × 100 _CH-π (CH···HC)_
**9** (Cl-PDI@MeOH)	−15.0	−7.5	2.918	3.120	1.07		0.65
**10** (Cl-PDI@EtOH)	−16.9	−8.5	2.828	2.641 (3.622)	0.88		0.46 (0.37)
**11** (Cl-PDI@PrOH)	−22.5	−11.3	2.842	3.075 (3.907)	0.76		0.81 (0.51)
**12** (Cl-PDI@BuOH)	−23.0	−11.5	2.787	2.765 (3.598)	1.00		0.75 (0.58)
**13** (Br-PDI@MeOH)	−15.5	−7.8	2.924	2.915	1.08		0.60
**14** (Br-PDI@EtOH)	−19.7	−9.9	3.070	2.863 (3.460)	0.84		0.64 (0.68)
**15** (Br-PDI@PrOH)	−22.8	−11.4	2.833	2.845 (3.912)	0.76		0.87 (0.51)
**16** (Br-PDI@BuOH)	−22.8	−11.4	2.781	2.794 (3.595)	0.98		0.71 (0.53)
**17** (PDI@MeOH)	−7.2	−3.6	2.904	2.783	1.22		0.77
**18** (PDI@EtOH)	−15.1	−7.6	2.878	2.873	1.13		0.57
**19** (PDI@PrOH)	−18.9	−9.5	3.066	2.829 (3.875)	0.87		0.71 (0.59)
**20** (PDI@BuOH)	−21.3	−10.7	2.960	2.704 (3.730)	1.04		0.77 (0.53)

*(1) Values given as the shortest distance between the VOC and the PDI moiety. In the case of the HC···CH interactions, only those cases where the C···C distance was lower than 4 Å were considered. *(2) The values corresponding to complexes **9** to **20** encompass the lp-π and CH-π interactions. The contribution of the CH-HC interactions between the two alcohol alkyl chains was subtracted from the complex (see Section 3 below).

## Data Availability

All data needed to reproduce the results derived from this study can be found in the ESI.

## Supplementary Materials

The following supporting information can be downloaded at: https://www.mdpi.com/article/10.3390/ijms25126436/s1.
