# Supplementary material for: Using Hybrid PDI-Fe3O4 Nanoparticles for Capturing Aliphatic Alcohols: Halogen Bonding vs. Lone Pair–π Interactions
Source: Int J Mol Sci. 2024 Jun 11;25(12):6436. doi: 10.3390/ijms25126436 (PMC11203483; doi:10.3390/ijms25126436)
Supplement: Supplementary file 1 [file ijms-25-06436-s001.zip › ijms-3010782-supplementary.pdf]

Article

Using Hybrid PDI-Fe_3O_4 Nanoparticles for Capturing Aliphatic Alcohols: Halogen Bonding vs. Lone Pair–π Interactions

María de las Nieves Piña, Alberto León, Antonio Frontera, Jeroni Morey * and Antonio Bauzá *

Department of Chemistry, Universitat de les Illes Balears, Ctra. de Valldemossa km. 7.5, 07122 Palma de Mallorca, Islas Baleares, Spain; neus.pinya@uib.es (M.d.l.N.P.); albertoperezleon@hotmail.com (A.L.); toni.frontera@uib.es (A.F.)

* Correspondence: jeroni.morey@uib.es (J.M.); antonio.bauza@uib.es (A.B.)

Electronic Supporting Information

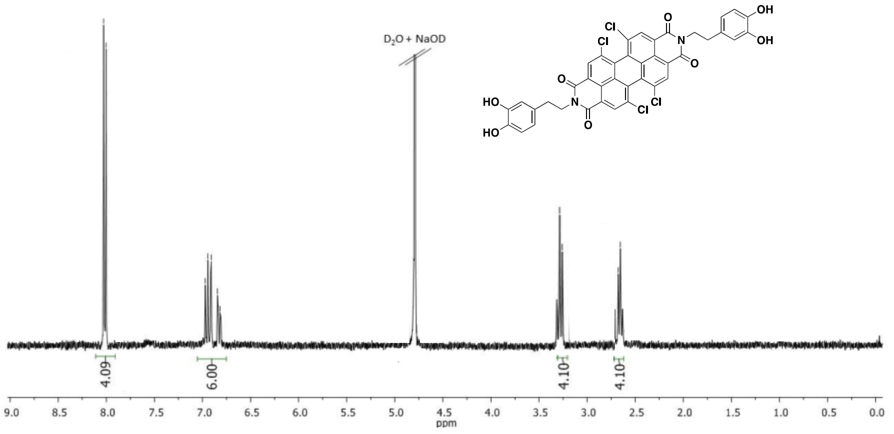

Figure S1. ^{1}H-RMN spectra of 2,9-bis(3,4-dihydroxyphenethyl)-1,6,7,12-tetrachloroperylene tetracarboxylic bisimide (Cl-PDI), D_2O/NaOD.

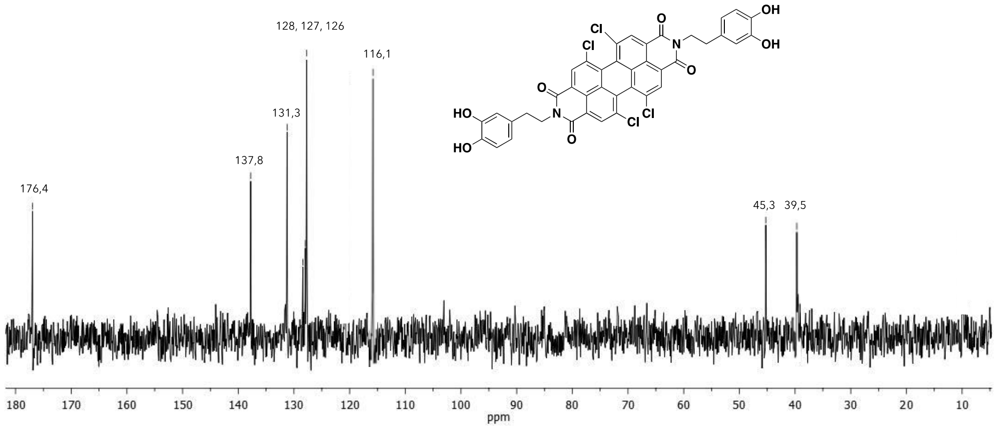

Figure S2. ^{13}C-RMN spectra of 2,9-bis(3,4-dihydroxyphenethyl)-1,6,7,12-tetrachloroperylene tetracarboxylic bisimide (Cl-PDI), D_2O/NaOD.

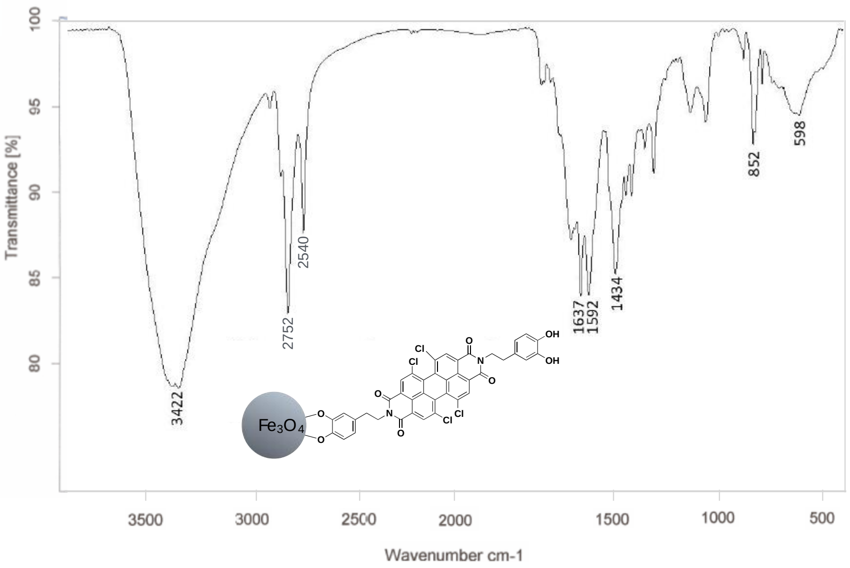

Figure S3. FT-IR spectra of functionalized magnetite nanoparticles Cl-PDI-NP, in KBr. It can be observed the corresponding bands related to magnetite and dopamine-Cl-PDI, indicating the presence of both in the final product.

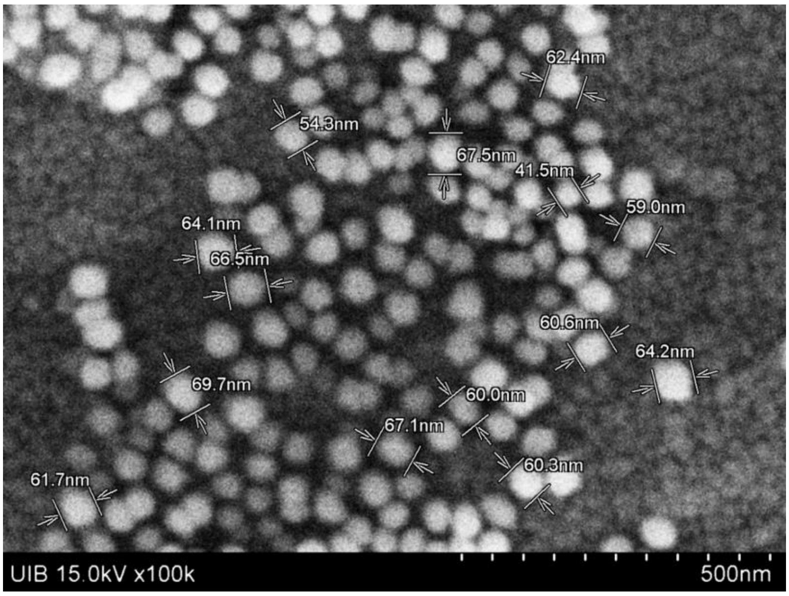

Figure S4. TEM micrograph of functionalized magnetite nanoparticles Cl-PDI-NP. It can be observed the nanoparticles are of same size (between 50 and 70 nm) and present a spherical shape.

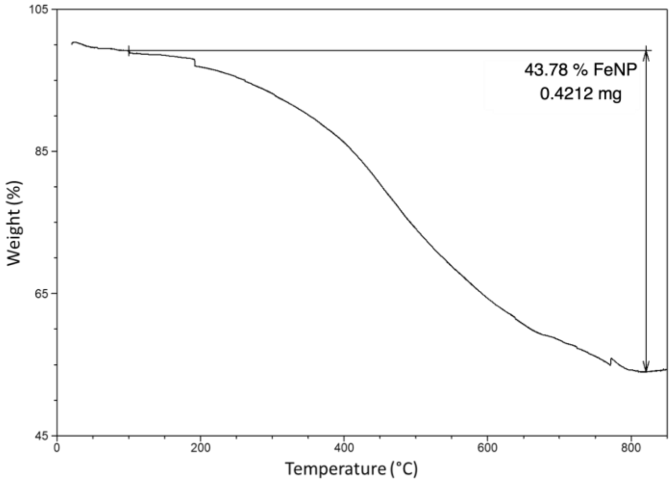

Figure S5. Thermogravimetric analysis (TGA) of magnetite nanoparticles Cl-PDI-NP. The percentage that appears in the TGA analysis corresponds to the loss of weight due to the volatilization of the PDI-NP coating, being a 43%. This corresponds to the NP surface coating percentage.

Calibration plots

In the following figures we represent the calibration plots obtained for each pair PDI-NP-VOC. All experiments were repeated at least 3 times in different sessions. Only those corresponding to one session are shown here, as a demonstration of the results obtained.

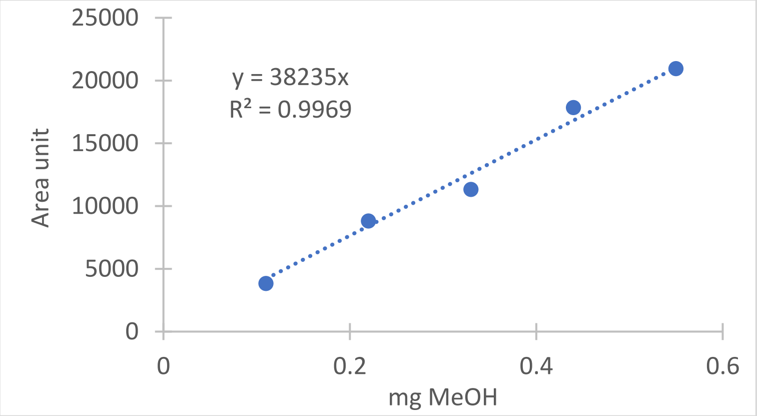

Figure S6. PDI-MeOH calibration plot. It can be observed a good linear correlation between the milligrams of methanol of each standard solution adsorbed by the PDI (x-axis) and the peak´s area of the chromatogram (y-axis).

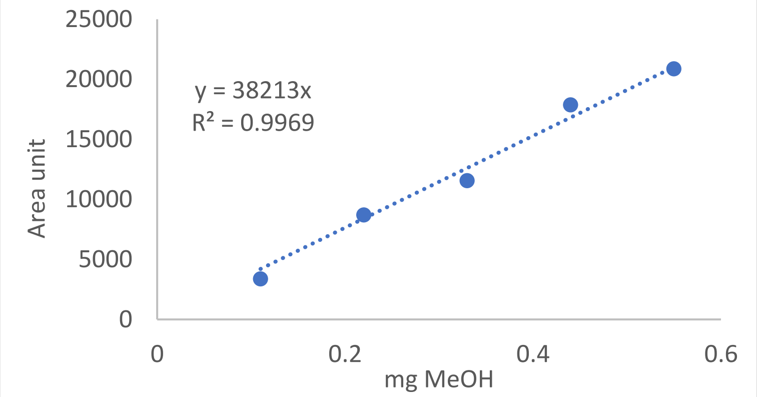

Figure S7. Cl-PDI-MeOH calibration plot. It can be observed a good linear correlation between the milligrams of methanol of each standard solution adsorbed by the Cl-PDI (x-axis) and the peak´s area of the chromatogram (y-axis).

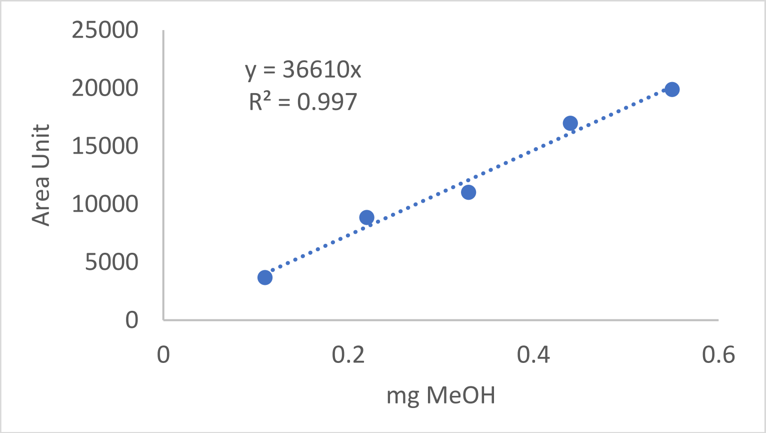

Figure S8. Br-PDI-MeOH calibration plot. It can be observed a good linear correlation between the milligrams of methanol of each standard solution adsorbed by the Br-PDI (x-axis) and the peak´s area of the chromatogram (y-axis).

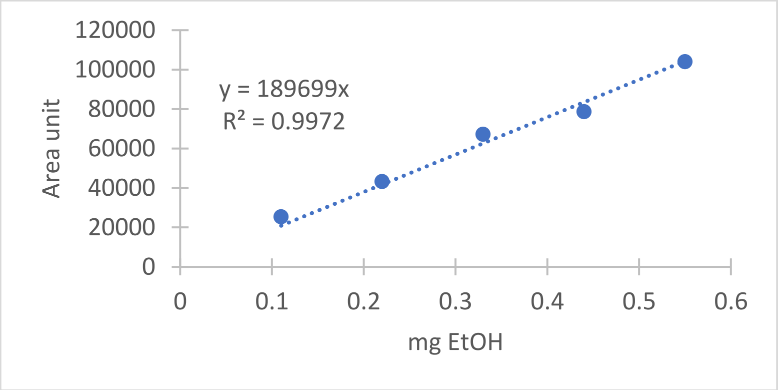

Figure S9. PDI-EtOH calibration plot. It can be observed a good linear correlation between the milligrams of ethanol of each standard solution adsorbed by the PDI (x-axis) and the peak´s area of the chromatogram (y-axis).

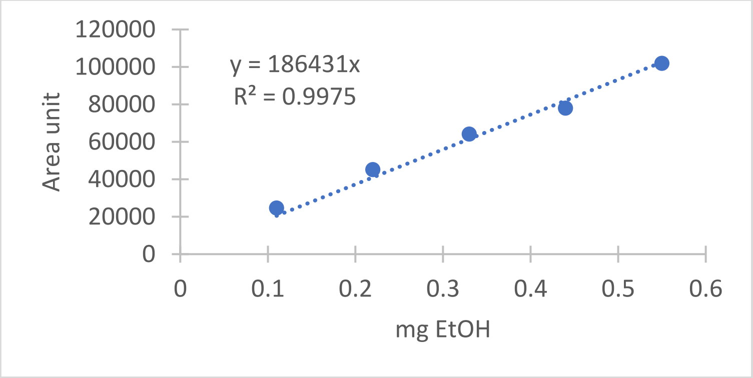

Figure S10. Cl-PDI-EtOH calibration plot. It can be observed a good linear correlation between the milligrams of ethanol of each standard solution adsorbed by the Cl-PDI (x-axis) and the peak´s area of the chromatogram (y-axis).

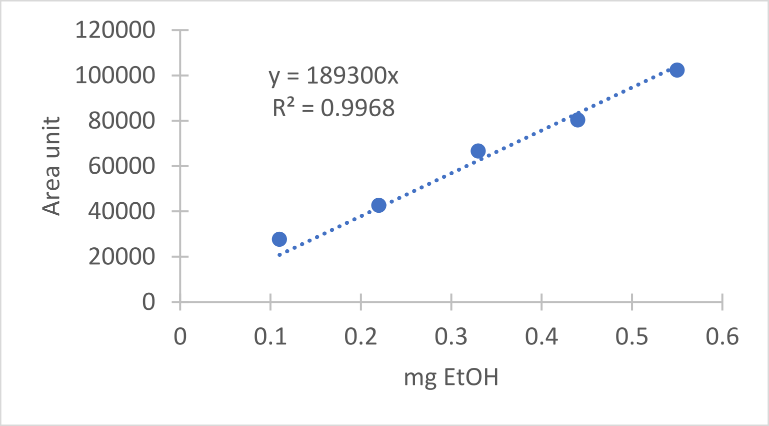

Figure S11. Br-PDI-EtOH calibration plot. It can be observed a good linear correlation between the milligrams of ethanol of each standard solution adsorbed by the Br-PDI (x-axis) and the peak´s area of the chromatogram (y-axis).

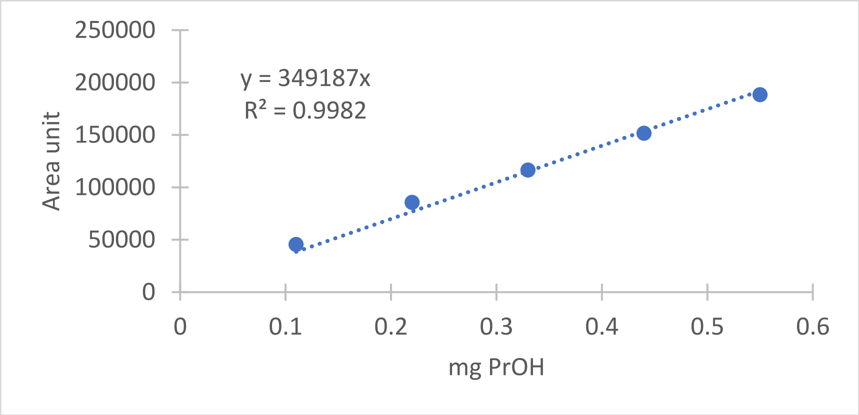

Figure S12. PDI-PrOH calibration plot. It can be observed a good linear correlation between the milligrams of propanol of each standard solution adsorbed by the PDI (x-axis) and the peak´s area of the chromatogram (y-axis).

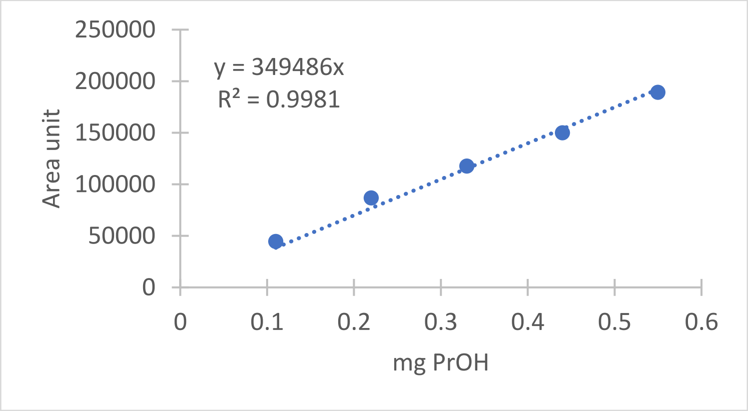

Figure S13. Cl-PDI-PrOH calibration plot. It can be observed a good linear correlation between the milligrams of propanol of each standard solution adsorbed by the Cl-PDI (x-axis) and the peak´s area of the chromatogram (y-axis).

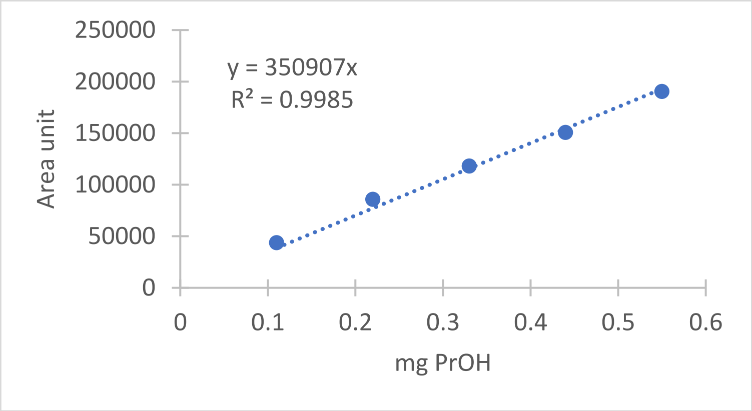

Figure S14. Br-PDI-PrOH calibration plot. It can be observed a good linear correlation between the milligrams of propanol of each standard solution adsorbed by the Br-PDI (x-axis) and the peak´s area of the chromatogram (y-axis).

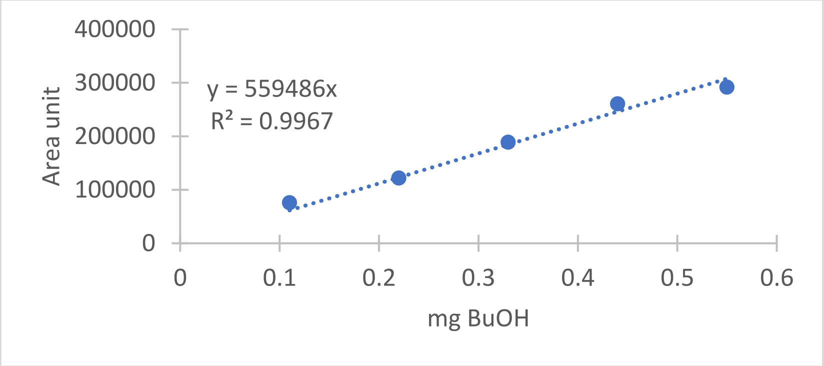

Figure S15. PDI-BuOH calibration plot. It can be observed a good linear correlation between the milligrams of butanol of each standard solution adsorbed by the PDI (x-axis) and the peak´s area of the chromatogram (y-axis).

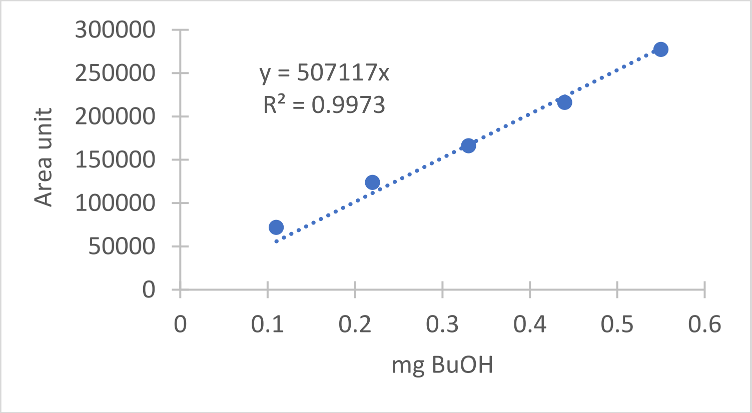

Figure S16. Cl-PDI-BuOH calibration plot. It can be observed a good linear correlation between the milligrams of butanol of each standard solution adsorbed by the Cl-PDI (x-axis) and the peak´s area of the chromatogram (y-axis).

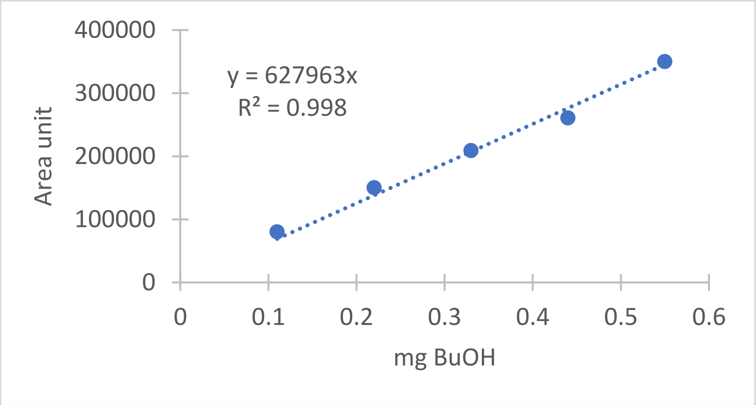

Figure S17. Br-PDI-BuOH calibration plot. It can be observed a good linear correlation between the milligrams of butanol of each standard solution adsorbed by the Br-PDI (x-axis) and the peak´s area of the chromatogram (y-axis).

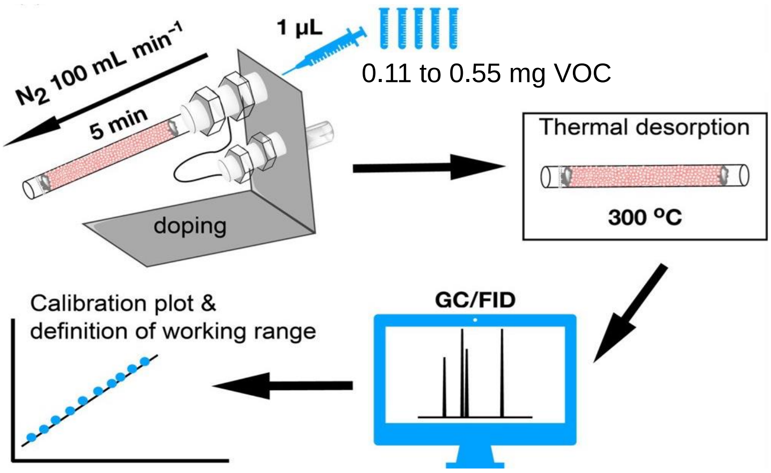

Figure S18. Methodology used to perform the calibration plots. The doping of the tube is carried out using 1 microliter of solution. 5 standard solutions were prepared, each one corresponding to a point of the calibration plot and containing different VOC quantities (0.11, 0.22, 0.33, 0.44 and 0.55 mg).

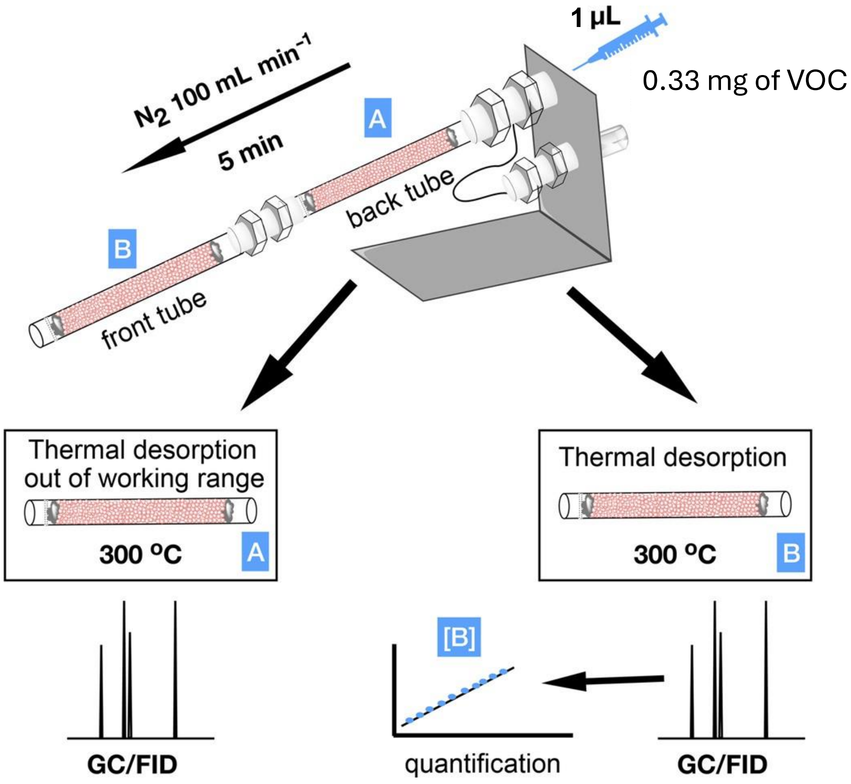

Figure S19. Schematic representation of the tubes connected in series. The quantitative analysis of front tube (B) allows us to quantify the amount of VOC adsorbed in the back tube (A). 1 microliter of the 0.33 mg standard solution was injected into the doping system. The VOC is retained in the back tube A, the leftover not retained by the tube A is recovered with the front tube, B . By analyzing front tube and comparing the quantity obtained with that initially injected (0.33 mg contained in 1 microliter of solution) we obtained the retention capacity of the material.

Cartesian coordinates of complexes 1 to 20

1

C	-2.9820772	2.0747651	-0.4340292
C	-2.6197453	0.7206527	-0.3329795
C	-1.3806993	0.3321644	0.2191994
C	-0.3993371	1.3604232	0.3951314
C	-0.7977379	2.7262700	0.3802093
C	-2.1095015	3.0677704	-0.0206567
C	-0.9817497	-1.0245967	0.5791394
C	0.9817497	1.0245967	0.5791394
C	1.3806993	-0.3321644	0.2191994
C	0.3993371	-1.3604232	0.3951314
C	2.6197453	-0.7206527	-0.3329795
C	1.8082200	2.0309113	1.1191773
C	1.3868383	3.3676141	1.1964522
C	0.1212023	3.7292635	0.7644431
H	2.0569179	4.1334518	1.5821733
H	-3.9571868	2.3523276	-0.8308677
C	-1.8082200	-2.0309113	1.1191773
C	-1.3868383	-3.3676141	1.1964522
C	-0.1212023	-3.7292635	0.7644431
C	0.7977379	-2.7262700	0.3802093
H	-2.0569179	-4.1334518	1.5821733
C	2.9820772	-2.0747651	-0.4340292
H	3.9571868	-2.3523276	-0.8308677
C	2.1095015	-3.0677704	-0.0206567
C	2.5349083	-4.4870657	-0.0589680
C	0.2712579	-5.1603383	0.7674553
C	-2.5349083	4.4870657	-0.0589680
C	-0.2712579	5.1603383	0.7674553
O	3.6515348	-4.8413089	-0.4233652
O	-0.4986100	-6.0515048	1.1127834
O	-3.6515348	4.8413089	-0.4233652
O	0.4986100	6.0515048	1.1127834
N	1.5860669	-5.4432703	0.3485776
N	-1.5860669	5.4432703	0.3485776
C	2.0290342	-6.8419315	0.3183794
H	2.9069860	-6.9634307	0.9629907
H	1.2030796	-7.4643382	0.6684348
H	2.3154975	-7.1162399	-0.7034507
C	-2.0290342	6.8419315	0.3183794
H	-2.3154975	7.1162399	-0.7034507
H	-2.9069860	6.9634307	0.9629907
H	-1.2030796	7.4643382	0.6684348
O	-4.7899294	-3.0911564	-2.0798050
O	4.7899294	3.0911564	-2.0798050
C	-3.8860221	-3.8568068	-1.2746377
H	-4.0740808	-3.7232581	-0.1960403
H	-3.9186723	-4.9324500	-1.5167366
C	3.8860221	3.8568068	-1.2746377
H	4.0740808	3.7232581	-0.1960403
H	3.9186723	4.9324500	-1.5167366
H	-5.6942029	-3.3505311	-1.8467350
H	5.6942029	3.3505311	-1.8467350
H	-2.8784625	-3.4816610	-1.4894143
H	2.8784625	3.4816610	-1.4894143
Cl	-3.7003790	-0.4352070	-1.0396449
Cl	-3.3494716	-1.6670219	1.8327843
Cl	3.7003790	0.4352070	-1.0396449
Cl	3.3494716	1.6670219	1.8327843

2

C	-3.2703551	1.5794914	-0.1401314
C	-2.7015750	0.2986036	-0.0338700
C	-1.4162490	0.1109436	0.5185699
C	-0.6079879	1.2807966	0.6897475
C	-1.2124094	2.5684449	0.6627218
C	-2.5612045	2.6995498	0.2609819
C	-0.8082820	-1.1657738	0.8773827
C	0.8082820	1.1657738	0.8773827
C	1.4162490	-0.1109436	0.5185699
C	0.6079879	-1.2807966	0.6897475
C	2.7015750	-0.2986036	-0.0338700
C	1.4659529	2.2899004	1.4162666
C	0.8434364	3.5460957	1.4785961
C	-0.4584413	3.7047289	1.0339233
H	1.3888711	4.4106653	1.8514581
H	-4.2778218	1.6990633	-0.5350673
C	-1.4659529	-2.2899004	1.4162666
C	-0.8434364	-3.5460957	1.4785961
C	0.4584413	-3.7047289	1.0339233
C	1.2124094	-2.5684449	0.6627218
H	-1.3888711	-4.4106653	1.8514581
C	3.2703551	-1.5794914	-0.1401314
H	4.2778218	-1.6990633	-0.5350673
C	2.5612045	-2.6995498	0.2609819
C	3.1991417	-4.0360730	0.2051278
C	1.0571911	-5.0607176	0.9951013
C	-3.1991417	4.0360730	0.2051278
C	-1.0571911	5.0607176	0.9951013
O	4.3595800	-4.2104418	-0.1537686
O	0.4244951	-6.0675272	1.2994809
O	-4.3595800	4.2104418	-0.1537686
O	-0.4244951	6.0675272	1.2994809
N	2.4021171	-5.1332399	0.5813765
N	-2.4021171	5.1332399	0.5813765
C	3.0466639	-6.4497905	0.5133300
H	3.9380994	-6.4558152	1.1504163
H	2.3240627	-7.1960557	0.8497716
H	3.3623700	-6.6538368	-0.5165906
C	-3.0466639	6.4497905	0.5133300
H	-3.3623700	6.6538368	-0.5165906
H	-3.9380994	6.4558152	1.1504163
H	-2.3240627	7.1960557	0.8497716
O	-4.6454002	-3.5280488	-2.1537741
O	4.6454002	3.5280488	-2.1537741
C	-3.9536567	-4.7778428	-1.9789868
H	-4.6588922	-5.5701464	-1.6679239
H	-3.4856638	-5.0988237	-2.9277338
C	3.9536567	4.7778428	-1.9789868
H	4.6588922	5.5701464	-1.6679239
H	3.4856638	5.0988237	-2.9277338
H	-5.3215895	-3.6428231	-2.8384923
H	5.3215895	3.6428231	-2.8384923
C	-2.8943993	-4.5702069	-0.9130852
H	-3.3615012	-4.2412125	0.0244021
H	-2.3404526	-5.4990560	-0.7211315
C	2.8943993	4.5702069	-0.9130852
H	3.3615012	4.2412125	0.0244021
H	2.3404526	5.4990560	-0.7211315
H	-2.1810407	-3.7956986	-1.2220005
H	2.1810407	3.7956986	-1.2220005
Cl	-3.5889766	-1.0112717	-0.7395243
Cl	3.0425847	2.1731903	2.1334257
Cl	3.5889766	1.0112717	-0.7395243

Cl	-3.0425847	-2.1731903	2.1334257

3

C	-3.6308337	0.2550251	-0.2317704
C	-2.6218012	-0.7197964	-0.1544408
C	-1.3557032	-0.4217206	0.3923835
C	-1.0400266	0.9636373	0.5760335
C	-2.0849780	1.9295005	0.5821189
C	-3.3904688	1.5505664	0.1945608
C	-0.3191205	-1.3841769	0.7493592
C	0.3191205	1.3841769	0.7493592
C	1.3557032	0.4217206	0.3923835
C	1.0400266	-0.9636373	0.5760335
C	2.6218012	0.7197964	-0.1544408
C	0.5178671	2.6719157	1.2882605
C	-0.5316594	3.5980683	1.3894946
C	-1.8092604	3.2581962	0.9767873
H	-0.3471470	4.5963345	1.7801895
H	-4.6141547	-0.0049553	-0.6200928
C	-0.5178671	-2.6719157	1.2882605
C	0.5316594	-3.5980683	1.3894946
C	1.8092604	-3.2581962	0.9767873
C	2.0849780	-1.9295005	0.5821189
H	0.3471470	-4.5963345	1.7801895
C	3.6308337	-0.2550251	-0.2317704
H	4.6141547	0.0049553	-0.6200928
C	3.3904688	-1.5505664	0.1945608
C	4.4872825	-2.5467090	0.1824102
C	2.8841929	-4.2801458	1.0110915
C	-4.4872825	2.5467090	0.1824102
C	-2.8841929	4.2801458	1.0110915
O	5.6323346	-2.2778583	-0.1682263
O	2.6829982	-5.4343286	1.3757279
O	-5.6323346	2.2778583	-0.1682263
O	-2.6829982	5.4343286	1.3757279
N	4.1617123	-3.8503603	0.5986973
N	-4.1617123	3.8503603	0.5986973
C	5.2622314	-4.8206637	0.5939398
H	6.0637083	-4.4726269	1.2557072
H	4.8676785	-5.7791112	0.9371381
H	5.6705137	-4.9089195	-0.4193882
C	-5.2622314	4.8206637	0.5939398
H	-5.6705137	4.9089195	-0.4193882
H	-6.0637083	4.4726269	1.2557072
H	-4.8676785	5.7791112	0.9371381
O	-3.2776735	-4.7423967	-2.4891525
O	3.2776735	4.7423967	-2.4891525
C	-3.7249129	-5.9443898	-1.8369513
H	-4.8287778	-5.9963721	-1.8304312
H	-3.3447014	-6.8351774	-2.3731651
C	3.7249129	5.9443898	-1.8369513
H	4.8287778	5.9963721	-1.8304312
H	3.3447014	6.8351774	-2.3731651
H	-3.6532945	-4.7165762	-3.3817123
H	3.6532945	4.7165762	-3.3817123
C	-3.1963995	-5.9372877	-0.4102892
H	-3.6126161	-5.0644469	0.1161336
H	-3.5885448	-6.8319338	0.0987611
C	3.1963995	5.9372877	-0.4102892
H	3.6126161	5.0644469	0.1161336
H	3.5885448	6.8319338	0.0987611
C	-1.6688460	-5.9089627	-0.3330155
H	-1.2732053	-4.9844414	-0.7719880

H	-1.2261300	-6.7566328	-0.8763513
C	1.6688460	5.9089627	-0.3330155
H	1.2732053	4.9844414	-0.7719880
H	1.2261300	6.7566328	-0.8763513
H	1.3282421	5.9636732	0.7085511
H	-1.3282421	-5.9636732	0.7085511
Cl	-2.9617908	-2.2521632	-0.8910475
Cl	-2.0362885	-3.1529493	1.9806767
Cl	2.9617908	2.2521632	-0.8910475
Cl	2.0362885	3.1529493	1.9806767

4

C	-1.5549383	-3.2910998	-0.8038131
C	-0.2790632	-2.7079610	-0.7151722
C	-0.0973760	-1.4175766	-0.1749096
C	-1.2752923	-0.6202502	0.0024365
C	-2.5555107	-1.2415623	0.0025961
C	-2.6761414	-2.5948385	-0.3867431
C	1.1740418	-0.7975651	0.1823665
C	-1.1740418	0.7975651	0.1823665
C	0.0973760	1.4175766	-0.1749096
C	1.2752923	0.6202502	0.0024365
C	0.2790632	2.7079610	-0.7151722
C	-2.3011979	1.4470099	0.7273347
C	-3.5492750	0.8098682	0.8115280
C	-3.6954644	-0.5018939	0.3909348
H	-4.4139278	1.3454857	1.1994006
H	-1.6684858	-4.3033918	-1.1875758
C	2.3011979	-1.4470099	0.7273347
C	3.5492750	-0.8098682	0.8115280
C	3.6954644	0.5018939	0.3909348
C	2.5555107	1.2415623	0.0025961
H	4.4139278	-1.3454857	1.1994006
C	1.5549383	3.2910998	-0.8038131
H	1.6684858	4.3033918	-1.1875758
C	2.6761414	2.5948385	-0.3867431
C	4.0021254	3.2560046	-0.4059730
C	5.0393117	1.1316628	0.4128193
C	-4.0021254	-3.2560046	-0.4059730
C	-5.0393117	-1.1316628	0.4128193
O	4.1638509	4.4210610	-0.7561833
O	6.0442411	0.5235199	0.7664540
O	-4.1638509	-4.4210610	-0.7561833
O	-6.0442411	-0.5235199	0.7664540
N	5.1004703	2.4791059	0.0032093
N	-5.1004703	-2.4791059	0.0032093
C	6.4045603	3.1521442	-0.0083174
H	6.3741801	4.0254478	0.6534108
H	7.1561234	2.4363882	0.3308026
H	6.6301199	3.5003995	-1.0228191
C	-6.4045603	-3.1521442	-0.0083174
H	-6.6301199	-3.5003995	-1.0228191
H	-6.3741801	-4.0254478	0.6534108
H	-7.1561234	-2.4363882	0.3308026
O	2.3699839	-6.5901544	-1.8326223
O	-2.3699839	6.5901544	-1.8326223
C	1.5248959	-7.6662167	-1.3852506
H	1.0382435	-8.1599326	-2.2463135
H	2.1199139	-8.4299121	-0.8487081
C	-1.5248959	7.6662167	-1.3852506
H	-1.0382435	8.1599326	-2.2463135
H	-2.1199139	8.4299121	-0.8487081
H	3.0874647	-6.9671099	-2.3635578

H	-3.0874647	6.9671099	-2.3635578
C	0.4710404	-7.0863697	-0.4533816
H	-0.0839727	-6.3084017	-1.0010817
H	-0.2549522	-7.8786373	-0.2097172
C	-0.4710404	7.0863697	-0.4533816
H	0.0839727	6.3084017	-1.0010817
H	0.2549522	7.8786373	-0.2097172
C	1.0540437	-6.4945817	0.8336614
H	1.8399684	-5.7753584	0.5644857
H	1.5482798	-7.2964753	1.4071224
C	-1.0540437	6.4945817	0.8336614
H	-1.8399684	5.7753584	0.5644857
H	-1.5482798	7.2964753	1.4071224
C	-0.0046042	-5.8103804	1.7004356
H	0.4257006	-5.4132877	2.6301944
H	-0.4638105	-4.9664750	1.1656243
H	-0.8110511	-6.5067098	1.9736535
C	0.0046042	5.8103804	1.7004356
H	-0.4257006	5.4132877	2.6301944
H	0.4638105	4.9664750	1.1656243
H	0.8110511	6.5067098	1.9736535
Cl	-2.1870788	3.0223739	1.4465885
Cl	-1.0229779	3.6191720	-1.4029196
Cl	1.0229779	-3.6191720	-1.4029196
Cl	2.1870788	-3.0223739	1.4465885

5

C	-3.4996694	0.9387448	-0.4541802
C	-2.6982336	-0.2110305	-0.3532508
C	-1.4097921	-0.1587246	0.2186404
C	-0.8375844	1.1421413	0.4030851
C	-1.6769748	2.2907711	0.3884939
C	-3.0235300	2.1667705	-0.0232990
C	-0.5747516	-1.2956078	0.5947085
C	0.5747516	1.2956078	0.5947085
C	1.4097921	0.1587246	0.2186404
C	0.8375844	-1.1421413	0.4030851
C	2.6982336	0.2110305	-0.3532508
C	1.0025353	2.5162472	1.1547197
C	0.1521714	3.6300355	1.2324585
C	-1.1569771	3.5439401	0.7831983
H	0.5117258	4.5758595	1.6336843
H	-4.5065212	0.8767077	-0.8632880
C	-1.0025353	-2.5162472	1.1547197
C	-0.1521714	-3.6300355	1.2324585
C	1.1569771	-3.5439401	0.7831983
C	1.6769748	-2.2907711	0.3884939
H	-0.5117258	-4.5758595	1.6336843
C	3.4996694	-0.9387448	-0.4541802
H	4.5065212	-0.8767077	-0.8632880
C	3.0235300	-2.1667705	-0.0232990
C	3.9072097	-3.3557652	-0.0598991
C	2.0118367	-4.7565738	0.7819684
C	-3.9072097	3.3557652	-0.0598991
C	-2.0118367	4.7565738	0.7819684
O	5.0760233	-3.3109164	-0.4303351
O	1.5928264	-5.8563783	1.1306100
O	-5.0760233	3.3109164	-0.4303351
O	-1.5928264	5.8563783	1.1306100
N	3.3423065	-4.5765508	0.3550127
N	-3.3423065	4.5765508	0.3550127
C	4.2347714	-5.7406777	0.3229411
H	5.1035548	-5.5555631	0.9649576

H	3.6716656	-6.6071939	0.6750649
H	4.5947221	-5.9015586	-0.6999201
C	-4.2347714	5.7406777	0.3229411
H	-4.5947221	5.9015586	-0.6999201
H	-5.1035548	5.5555631	0.9649576
H	-3.6716656	6.6071939	0.6750649
O	-3.6230946	-4.7217414	-1.9792311
O	3.6230946	4.7217414	-1.9792311
C	-2.5989126	-5.3328834	-1.1869906
H	-3.0036077	-6.0795007	-0.4832635
H	-1.8172240	-5.8059682	-1.8048435
C	2.5989126	5.3328834	-1.1869906
H	3.0036077	6.0795007	-0.4832635
H	1.8172240	5.8059682	-1.8048435
H	-4.0491826	-5.4066525	-2.5156619
H	4.0491826	5.4066525	-2.5156619
Br	3.3674062	1.7838746	-1.1848483
Br	2.6972609	2.7075310	2.0035290
Br	-3.3674062	-1.7838746	-1.1848483
Br	-2.6972609	-2.7075310	2.0035290
H	-2.1365076	-4.5277140	-0.6040612
H	2.1365076	4.5277140	-0.6040612

6

C	-3.5839990	-0.4755722	-0.2040644
C	-2.4044383	-1.2310207	-0.0915069
C	-1.2401139	-0.6875065	0.4901499
C	-1.2114650	0.7327118	0.6732176
C	-2.4253663	1.4730941	0.6463574
C	-3.6176249	0.8430690	0.2229651
C	-0.0333145	-1.4160039	0.8696508
C	0.0333145	1.4160039	0.8696508
C	1.2401139	0.6875065	0.4901499
C	1.2114650	-0.7327118	0.6732176
C	2.4044383	1.2310207	-0.0915069
C	-0.0415648	2.7060263	1.4298274
C	-1.2534607	3.4092740	1.5011014
C	-2.4251009	2.8312581	1.0371118
H	-1.2842612	4.4232584	1.8943870
H	-4.4862474	-0.9183475	-0.6224856
C	0.0415648	-2.7060263	1.4298274
C	1.2534607	-3.4092740	1.5011014
C	2.4251009	-2.8312581	1.0371118
C	2.4253663	-1.4730941	0.6463574
H	1.2842612	-4.4232584	1.8943870
C	3.5839990	0.4755722	-0.2040644
H	4.4862474	0.9183475	-0.6224856
C	3.6176249	-0.8430690	0.2229651
C	4.8860127	-1.6071351	0.1674711
C	3.6727152	-3.6314475	1.0035749
C	-4.8860127	1.6071351	0.1674711
C	-3.6727152	3.6314475	1.0035749
O	5.9465629	-1.1198897	-0.2118104
O	3.7043864	-4.8149140	1.3284498
O	-5.9465629	1.1198897	-0.2118104
O	-3.7043864	4.8149140	1.3284498
N	4.8307336	-2.9551243	0.5704208
N	-4.8307336	2.9551243	0.5704208
C	6.0963015	-3.6954828	0.5103011
H	6.8414139	-3.2017050	1.1444464
H	5.9076361	-4.7138881	0.8559425
H	6.4735051	-3.6991550	-0.5189569
C	-6.0963015	3.6954828	0.5103011

H	-6.4735051	3.6991550	-0.5189569
H	-6.8414139	3.2017050	1.1444464
H	-5.9076361	4.7138881	0.8559425
O	-1.7163881	-5.6291151	-2.2493452
O	1.7163881	5.6291151	-2.2493452
C	-0.4950840	-6.3162173	-1.9150428
H	-0.7160922	-7.3286522	-1.5308889
H	0.1416024	-6.4275898	-2.8115599
C	0.4950840	6.3162173	-1.9150428
H	0.7160922	7.3286522	-1.5308889
H	-0.1416024	6.4275898	-2.8115599
H	-2.1938161	-6.1507547	-2.9119866
H	2.1938161	6.1507547	-2.9119866
Br	2.3962945	2.9359998	-0.9343960
Br	1.4523067	3.5342310	2.2728138
Br	-2.3962945	-2.9359998	-0.9343960
Br	-1.4523067	-3.5342310	2.2728138
C	0.2217229	-5.4993597	-0.8571540
H	-0.4116646	-5.3915802	0.0329636
H	1.1634456	-5.9797993	-0.5601932
C	-0.2217229	5.4993597	-0.8571540
H	0.4116646	5.3915802	0.0329636
H	-1.1634456	5.9797993	-0.5601932
H	0.4475982	-4.4929217	-1.2317617
H	-0.4475982	4.4929217	-1.2317617

7

C	-2.9049448	-2.1690072	-0.2756270
C	-1.5054951	-2.2498881	-0.1838734
C	-0.7483269	-1.2051312	0.3849393
C	-1.4152886	0.0493879	0.5718902
C	-2.8370839	0.0994280	0.5657870
C	-3.5742221	-1.0362992	0.1604089
C	0.6617450	-1.2545222	0.7590930
C	-0.6617450	1.2545222	0.7590930
C	0.7483269	1.2051312	0.3849393
C	1.4152886	-0.0493879	0.5718902
C	1.5054951	2.2498881	-0.1838734
C	-1.3545852	2.3481580	1.3157077
C	-2.7544631	2.3673867	1.4037909
C	-3.4982215	1.2831663	0.9640013
H	-3.2746051	3.2348998	1.8043567
H	-3.4810457	-2.9986902	-0.6819863
C	1.3545852	-2.3481580	1.3157077
C	2.7544631	-2.3673867	1.4037909
C	3.4982215	-1.2831663	0.9640013
C	2.8370839	-0.0994280	0.5657870
H	3.2746051	-3.2348998	1.8043567
C	2.9049448	2.1690072	-0.2756270
H	3.4810457	2.9986902	-0.6819863
C	3.5742221	1.0362992	0.1604089
C	5.0551134	0.9971736	0.1347452
C	4.9798096	-1.3579700	0.9786692
C	-5.0551134	-0.9971736	0.1347452
C	-4.9798096	1.3579700	0.9786692
O	5.7443326	1.9456220	-0.2282769
O	5.5846882	-2.3627230	1.3399322
O	-5.7443326	-1.9456220	-0.2282769
O	-5.5846882	2.3627230	1.3399322
N	5.6631460	-0.2013724	0.5516098
N	-5.6631460	0.2013724	0.5516098
C	7.1299761	-0.2199426	0.5285289
H	7.5191456	0.5688143	1.1826222

H	7.4598796	-1.2027579	0.8708778
H	7.4844884	-0.0238723	-0.4900223
C	-7.1299761	0.2199426	0.5285289
H	-7.4844884	0.0238723	-0.4900223
H	-7.5191456	-0.5688143	1.1826222
H	-7.4598796	1.2027579	0.8708778
O	0.7690932	-5.8644692	-2.4893012
O	-0.7690932	5.8644692	-2.4893012
C	1.1914312	-7.0459798	-1.7805853
H	0.3842469	-7.8000960	-1.7693529
H	2.0705842	-7.4923585	-2.2825541
C	-1.1914312	7.0459798	-1.7805853
H	-0.3842469	7.8000960	-1.7693529
H	-2.0705842	7.4923585	-2.2825541
H	0.4501454	-6.1257654	-3.3659813
H	-0.4501454	6.1257654	-3.3659813
Br	0.6871432	3.7429846	-1.0387563
Br	-0.4510679	3.7993364	2.1588090
Br	-0.6871432	-3.7429846	-1.0387563
Br	0.4510679	-3.7993364	2.1588090
C	1.5528073	-6.6502764	-0.3567161
H	0.6533577	-6.2559014	0.1407228
H	1.8370059	-7.5675290	0.1832426
C	-1.5528073	6.6502764	-0.3567161
H	-0.6533577	6.2559014	0.1407228
H	-1.8370059	7.5675290	0.1832426
C	2.6796803	-5.6189268	-0.2798907
H	2.3805898	-4.6762951	-0.7555368
H	3.5872582	-5.9795650	-0.7854251
C	-2.6796803	5.6189268	-0.2798907
H	-2.3805898	4.6762951	-0.7555368
H	-3.5872582	5.9795650	-0.7854251
H	-2.9399741	5.4035709	0.7641512
H	2.9399741	-5.4035709	0.7641512

8

C	-2.4681795	-2.6591043	-0.5955621
C	-1.0766954	-2.4830277	-0.5132245
C	-0.5173416	-1.3199092	0.0532215
C	-1.4011665	-0.2078755	0.2454432
C	-2.8079712	-0.4177808	0.2520065
C	-3.3289490	-1.6681526	-0.1515242
C	0.8777579	-1.1133860	0.4296655
C	-0.8777579	1.1133860	0.4296655
C	0.5173416	1.3199092	0.0532215
C	1.4011665	0.2078755	0.2454432
C	1.0766954	2.4830277	-0.5132245
C	-1.7521085	2.0627287	0.9950817
C	-3.1321954	1.8276250	1.0940836
C	-3.6698567	0.6249946	0.6607169
H	-3.7971962	2.5872468	1.5008460
H	-2.8864021	-3.5794948	-0.9993206
C	1.7521085	-2.0627287	0.9950817
C	3.1321954	-1.8276250	1.0940836
C	3.6698567	-0.6249946	0.6607169
C	2.8079712	0.4177808	0.2520065
H	3.7971962	-2.5872468	1.5008460
C	2.4681795	2.6591043	-0.5955621
H	2.8864021	3.5794948	-0.9993206
C	3.3289490	1.6681526	-0.1515242
C	4.7921437	1.8995672	-0.1674072
C	5.1398525	-0.4249050	0.6940325
C	-4.7921437	-1.8995672	-0.1674072

C	-5.1398525	0.4249050	0.6940325
O	5.2994018	2.9550099	-0.5352105
O	5.9144614	-1.2982262	1.0717085
O	-5.2994018	-2.9550099	-0.5352105
O	-5.9144614	1.2982262	1.0717085
N	5.6042068	0.8356839	0.2656183
N	-5.6042068	-0.8356839	0.2656183
C	7.0498199	1.0865906	0.2588680
H	7.2772141	1.9461986	0.8996123
H	7.5496125	0.1880423	0.6264110
H	7.3778231	1.3239931	-0.7597756
C	-7.0498199	-1.0865906	0.2588680
H	-7.3778231	-1.3239931	-0.7597756
H	-7.2772141	-1.9461986	0.8996123
H	-7.5496125	-0.1880423	0.6264110
O	1.0681144	-6.7229166	-2.1076812
O	-1.0681144	6.7229166	-2.1076812
C	0.1998064	-7.8399424	-1.8310807
H	-0.2482412	-8.2194298	-2.7672085
H	0.7712269	-8.6662962	-1.3674815
C	-0.1998064	7.8399424	-1.8310807
H	0.2482412	8.2194298	-2.7672085
H	-0.7712269	8.6662962	-1.3674815
H	1.8079299	-7.0352083	-2.6495601
H	-1.8079299	7.0352083	-2.6495601
Br	0.0243064	3.8268703	-1.3512436
Br	-1.1138800	3.6521803	1.8291550
Br	-0.0243064	-3.8268703	-1.3512436
Br	1.1138800	-3.6521803	1.8291550
C	-0.8920098	-7.3659320	-0.8834024
H	-1.4055715	-6.5109110	-1.3509761
H	-1.6432445	-8.1661393	-0.7837628
C	0.8920098	7.3659320	-0.8834024
H	1.4055715	6.5109110	-1.3509761
H	1.6432445	8.1661393	-0.7837628
C	-0.3685303	-6.9623752	0.4993909
H	0.4961507	-6.2962912	0.3703884
H	0.0040213	-7.8592949	1.0212933
C	0.3685303	6.9623752	0.4993909
H	-0.4961507	6.2962912	0.3703884
H	-0.0040213	7.8592949	1.0212933
C	-1.4319603	-6.2633674	1.3481695
H	-1.0553070	-6.0165354	2.3507089
H	-1.7494421	-5.3225940	0.8747103
H	-2.3262628	-6.8913972	1.4732896
C	1.4319603	6.2633674	1.3481695
H	1.0553070	6.0165354	2.3507089
H	1.7494421	5.3225940	0.8747103
H	2.3262628	6.8913972	1.4732896

9

C	-2.6337635	-2.4912347	-0.2108912
C	-1.2356069	-2.4072566	-0.1047815
C	-0.6013044	-1.2832183	0.4608855
C	-1.4134539	-0.1171266	0.6457612
C	-2.8302092	-0.2326738	0.6280491
C	-3.4314675	-1.4419834	0.2138522
C	0.8068138	-1.1685074	0.8229280
C	-0.8068138	1.1685074	0.8229280
C	0.6013044	1.2832183	0.4608855
C	1.4134539	0.1171266	0.6457612
C	1.2356069	2.4072566	-0.1047815
C	-1.6283066	2.1880509	1.3442691

C	-3.0253449	2.0488845	1.4038755
C	-3.6280979	0.8760619	0.9837414
H	-3.6446228	2.8720934	1.7560274
H	-3.1017228	-3.3842416	-0.6214097
C	1.6283066	-2.1880509	1.3442691
C	3.0253449	-2.0488845	1.4038755
C	3.6280979	-0.8760619	0.9837414
C	2.8302092	0.2326738	0.6280491
H	3.6446228	-2.8720934	1.7560274
C	2.6337635	2.4912347	-0.2108912
H	3.1017228	3.3842416	-0.6214097
C	3.4314675	1.4419834	0.2138522
C	4.9091297	1.5749571	0.1575836
C	5.1060432	-0.7933845	0.9030648
C	-4.9091297	-1.5749571	0.1575836
C	-5.1060432	0.7933845	0.9030648
O	5.4607913	2.6017691	-0.2237576
O	5.8336117	-1.7604811	1.1157810
O	-5.4607913	-2.6017691	-0.2237576
O	-5.8336117	1.7604811	1.1157810
N	5.6523125	0.4540609	0.5708794
N	-5.6523125	-0.4540609	0.5708794
C	7.1146463	0.5295173	0.4950656
H	7.4001811	1.5830129	0.4652275
H	7.5434309	0.0242156	1.3654354
H	7.4705785	0.0257137	-0.4129715
C	-7.1146463	-0.5295173	0.4950656
H	-7.4705785	-0.0257137	-0.4129715
H	-7.4001811	-1.5830129	0.4652275
H	-7.5434309	-0.0242156	1.3654354
O	4.4004261	-1.1568045	-1.9254154
O	-4.4004261	1.1568045	-1.9254154
C	3.0348647	-1.4186605	-2.2691178
H	2.5536497	-0.4425870	-2.4057876
H	2.9459100	-1.9888518	-3.2086758
C	-3.0348647	1.4186605	-2.2691178
H	-2.5536497	0.4425870	-2.4057876
H	-2.9459100	1.9888518	-3.2086758
H	4.8217757	-1.9974382	-1.6871482
H	-4.8217757	1.9974382	-1.6871482
Cl	-0.3278453	-3.6996100	-0.8328522
Cl	0.9644838	-3.6335135	2.0420579
Cl	-0.9644838	3.6335135	2.0420579
Cl	0.3278453	3.6996100	-0.8328522
H	-2.4980456	1.9595874	-1.4716760
H	2.4980456	-1.9595874	-1.4716760

10

C	-2.6782408	-2.3940413	0.1821053
C	-1.2827404	-2.3612961	0.3405188
C	-0.6308329	-1.2668904	0.9416351
C	-1.4148985	-0.0816063	1.1286395
C	-2.8325307	-0.1531262	1.0745596
C	-3.4558398	-1.3289488	0.6027873
C	0.7782592	-1.1886959	1.3083066
C	-0.7782592	1.1886959	1.3083066
C	0.6308329	1.2668904	0.9416351
C	1.4148985	0.0816063	1.1286395
C	1.2827404	2.3612961	0.3405188
C	-1.5775029	2.2315756	1.8160150
C	-2.9776817	2.1271012	1.8658550
C	-3.6052771	0.9739196	1.4277776
H	-3.5799338	2.9627736	2.2182949

H	-3.1594305	-3.2559415	-0.2767148
C	1.5775029	-2.2315756	1.8160150
C	2.9776817	-2.1271012	1.8658550
C	3.6052771	-0.9739196	1.4277776
C	2.8325307	0.1531262	1.0745596
H	3.5799338	-2.9627736	2.2182949
C	2.6782408	2.3940413	0.1821053
H	3.1594305	3.2559415	-0.2767148
C	3.4558398	1.3289488	0.6027873
C	4.9305751	1.3914115	0.4416251
C	5.0843789	-0.9268668	1.3417675
C	-4.9305751	-1.3914115	0.4416251
C	-5.0843789	0.9268668	1.3417675
O	5.4917908	2.3632572	-0.0559593
O	5.7965176	-1.8803781	1.6412841
O	-5.4917908	-2.3632572	-0.0559593
O	-5.7965176	1.8803781	1.6412841
N	5.6536935	0.2744031	0.8905575
N	-5.6536935	-0.2744031	0.8905575
C	7.1139895	0.2897491	0.7591979
H	7.4247559	1.3138682	0.5430824
H	7.5624193	-0.0740567	1.6890676
H	7.4210964	-0.3751573	-0.0578134
C	-7.1139895	-0.2897491	0.7591979
H	-7.4210964	0.3751573	-0.0578134
H	-7.4247559	-1.3138682	0.5430824
H	-7.5624193	0.0740567	1.6890676
O	4.0111917	-0.8807573	-1.6354597
O	-4.0111917	0.8807573	-1.6354597
C	3.3168716	-0.0883245	-2.6133363
H	3.6490815	0.9424860	-2.4273480
H	3.6455485	-0.3620238	-3.6320901
C	-3.3168716	0.0883245	-2.6133363
H	-3.6490815	-0.9424860	-2.4273480
H	-3.6455485	0.3620238	-3.6320901
H	3.7115539	-1.7998375	-1.7205287
H	-3.7115539	1.7998375	-1.7205287
C	1.8009531	-0.1905079	-2.4974626
H	1.4578369	0.1453083	-1.5116553
H	1.3107977	0.4312696	-3.2595209
C	-1.8009531	0.1905079	-2.4974626
H	-1.4578369	-0.1453083	-1.5116553
H	-1.3107977	-0.4312696	-3.2595209
Cl	-0.3967049	-3.6682585	-0.3891437
Cl	0.8788794	-3.6653569	2.5051215
Cl	-0.8788794	3.6653569	2.5051215
Cl	0.3967049	3.6682585	-0.3891437
H	-1.4594452	1.2269880	-2.6411654
H	1.4594452	-1.2269880	-2.6411654

11

C	-2.6515052	-2.4934063	0.4357670
C	-1.2528739	-2.4200833	0.5462828
C	-0.6088148	-1.2853496	1.0826324
C	-1.4149190	-0.1114470	1.2517813
C	-2.8327482	-0.2160925	1.2212990
C	-3.4420500	-1.4262480	0.8234537
C	0.8020626	-1.1721897	1.4328950
C	-0.8020626	1.1721897	1.4328950
C	0.6088148	1.2853496	1.0826324
C	1.4149190	0.1114470	1.2517813
C	1.2528739	2.4200833	0.5462828
C	-1.6229498	2.1957814	1.9486342

C	-3.0215117	2.0711489	1.9809709
C	-3.6275262	0.9022304	1.5542683
H	-3.6396215	2.8967144	2.3298297
H	-3.1245700	-3.3892299	0.0376183
C	1.6229498	-2.1957814	1.9486342
C	3.0215117	-2.0711489	1.9809709
C	3.6275262	-0.9022304	1.5542683
C	2.8327482	0.2160925	1.2212990
H	3.6396215	-2.8967144	2.3298297
C	2.6515052	2.4934063	0.4357670
H	3.1245700	3.3892299	0.0376183
C	3.4420500	1.4262480	0.8234537
C	4.9193851	1.5365551	0.7204412
C	5.1076097	-0.8219618	1.4899782
C	-4.9193851	-1.5365551	0.7204412
C	-5.1076097	0.8219618	1.4899782
O	5.4700994	2.5500745	0.3023251
O	5.8305189	-1.7839890	1.7339631
O	-5.4700994	-2.5500745	0.3023251
O	-5.8305189	1.7839890	1.7339631
N	5.6590908	0.4163843	1.1339598
N	-5.6590908	-0.4163843	1.1339598
C	7.1200295	0.4711391	1.0258734
H	7.4153363	1.5180131	0.9313241
H	7.5623676	0.0108181	1.9144501
H	7.4475126	-0.0891831	0.1407553
C	-7.1200295	-0.4711391	1.0258734
H	-7.4475126	0.0891831	0.1407553
H	-7.4153363	-1.5180131	0.9313241
H	-7.5623676	-0.0108181	1.9144501
O	4.4138568	-0.6392990	-1.5169818
O	-4.4138568	0.6392990	-1.5169818
C	3.6529414	-0.1330614	-2.6268920
H	4.0826636	0.8551706	-2.8392441
H	3.8122809	-0.7661758	-3.5196278
C	-3.6529414	0.1330614	-2.6268920
H	-4.0826636	-0.8551706	-2.8392441
H	-3.8122809	0.7661758	-3.5196278
H	4.1549881	-1.5619541	-1.3664711
H	-4.1549881	1.5619541	-1.3664711
C	2.1568754	-0.0086947	-2.3337416
H	2.0168789	0.7061296	-1.5101968
H	1.6718624	0.4351493	-3.2179534
C	-2.1568754	0.0086947	-2.3337416
H	-2.0168789	-0.7061296	-1.5101968
H	-1.6718624	-0.4351493	-3.2179534
C	1.4935226	-1.3398949	-1.9841086
H	1.9366901	-1.7867687	-1.0828917
H	1.5862127	-2.0671128	-2.8040404
C	-1.4935226	1.3398949	-1.9841086
H	-1.9366901	1.7867687	-1.0828917
H	-1.5862127	2.0671128	-2.8040404
Cl	-0.3684376	-3.7442043	-0.1498366
Cl	0.9588544	-3.6220918	2.6850393
Cl	-0.9588544	3.6220918	2.6850393
Cl	0.3684376	3.7442043	-0.1498366
H	0.4243566	-1.2130307	-1.7715562
H	-0.4243566	1.2130307	-1.7715562

12

C	-3.4579373	-1.1595353	0.6224135
C	-2.1568275	-1.6792052	0.7226101
C	-1.0903313	-0.9152700	1.2403116

C	-1.3318342	0.4880249	1.4174176
C	-2.6680238	0.9749658	1.4342394
C	-3.7294252	0.1320333	1.0386258
C	0.2397900	-1.4004572	1.5948588
C	-0.2397900	1.4004572	1.5948588
C	1.0903313	0.9152700	1.2403116
C	1.3318342	-0.4880249	1.4174176
C	2.1568275	1.6792052	0.7226101
C	-0.5569403	2.6580489	2.1486628
C	-1.8830779	3.1135589	2.2364018
C	-2.9274026	2.3070980	1.8213197
H	-2.0980157	4.1066566	2.6272954
H	-4.2668050	-1.7746310	0.2323673
C	0.5569403	-2.6580489	2.1486628
C	1.8830779	-3.1135589	2.2364018
C	2.9274026	-2.3070980	1.8213197
C	2.6680238	-0.9749658	1.4342394
H	2.0980157	-4.1066566	2.6272954
C	3.4579373	1.1595353	0.6224135
H	4.2668050	1.7746310	0.2323673
C	3.7294252	-0.1320333	1.0386258
C	5.1230089	-0.6415819	0.9873206
C	4.3148767	-2.8323288	1.8361379
C	-5.1230089	0.6415819	0.9873206
C	-4.3148767	2.8323288	1.8361379
O	6.0463278	0.0267650	0.5305477
O	4.5748872	-3.9937669	2.1343247
O	-6.0463278	-0.0267650	0.5305477
O	-4.5748872	3.9937669	2.1343247
N	5.3353088	-1.9306780	1.4967143
N	-5.3353088	1.9306780	1.4967143
C	6.6963811	-2.4713903	1.4429750
H	7.3993516	-1.6354378	1.4451856
H	6.8506368	-3.1262662	2.3047812
H	6.8259238	-3.0580443	0.5240722
C	-6.6963811	2.4713903	1.4429750
H	-6.8259238	3.0580443	0.5240722
H	-7.3993516	1.6354378	1.4451856
H	-6.8506368	3.1262662	2.3047812
O	4.0023269	-2.7047046	-1.0630774
O	-4.0023269	2.7047046	-1.0630774
C	3.7411663	-1.7873368	-2.1387396
H	4.4560676	-0.9663662	-1.9912419
H	3.9754938	-2.2645425	-3.1087083
C	-3.7411663	1.7873368	-2.1387396
H	-4.4560676	0.9663662	-1.9912419
H	-3.9754938	2.2645425	-3.1087083
H	3.4475939	-3.4905960	-1.1869185
H	-3.4475939	3.4905960	-1.1869185
C	2.3099828	-1.2538901	-2.1580541
H	2.1283560	-0.6482372	-1.2576232
H	2.2183955	-0.5669410	-3.0157076
C	-2.3099828	1.2538901	-2.1580541
H	-2.1283560	0.6482372	-1.2576232
H	-2.2183955	0.5669410	-3.0157076
C	1.2449762	-2.3507177	-2.2620932
H	1.2353864	-2.9443679	-1.3321639
H	1.5140333	-3.0443822	-3.0771477
C	-1.2449762	2.3507177	-2.2620932
H	-1.2353864	2.9443679	-1.3321639
H	-1.5140333	3.0443822	-3.0771477
C	-0.1567688	-1.7924252	-2.5075353
H	-0.9043573	-2.5908738	-2.5911384

H	-0.4667521	-1.1342089	-1.6845865
H	-0.1875344	-1.1983879	-3.4312863
C	0.1567688	1.7924252	-2.5075353
H	0.9043573	2.5908738	-2.5911384
H	0.4667521	1.1342089	-1.6845865
H	0.1875344	1.1983879	-3.4312863
Cl	-1.9230768	-3.2736529	0.0736593
Cl	-0.6472231	-3.6652549	2.8937798
Cl	0.6472231	3.6652549	2.8937798
Cl	1.9230768	3.2736529	0.0736593

13

C	-2.5695242	-2.5362126	-0.2511251
C	-1.1747518	-2.4211320	-0.1354523
C	-0.5693290	-1.2952612	0.4545330
C	-1.4088342	-0.1495185	0.6469466
C	-2.8218573	-0.2966122	0.6244736
C	-3.3934046	-1.5132009	0.1911637
C	0.8316489	-1.1482263	0.8355157
C	-0.8316489	1.1482263	0.8355157
C	0.5693290	1.2952612	0.4545330
C	1.4088342	0.1495185	0.6469466
C	1.1747518	2.4211320	-0.1354523
C	-1.6733473	2.1368607	1.3799090
C	-3.0664740	1.9667982	1.4401734
C	-3.6438478	0.7891121	0.9949197
H	-3.7081488	2.7645289	1.8101663
H	-3.0208997	-3.4282809	-0.6818762
C	1.6733473	-2.1368607	1.3799090
C	3.0664740	-1.9667982	1.4401734
C	3.6438478	-0.7891121	0.9949197
C	2.8218573	0.2966122	0.6244736
H	3.7081488	-2.7645289	1.8101663
C	2.5695242	2.5362126	-0.2511251
H	3.0208997	3.4282809	-0.6818762
C	3.3934046	1.5132009	0.1911637
C	4.8674783	1.6780401	0.1281257
C	5.1191617	-0.6753221	0.9071594
C	-4.8674783	-1.6780401	0.1281257
C	-5.1191617	0.6753221	0.9071594
O	5.3950872	2.7113686	-0.2692985
O	5.8688501	-1.6225853	1.1314479
O	-5.3950872	-2.7113686	-0.2692985
O	-5.8688501	1.6225853	1.1314479
N	5.6363095	0.5789663	0.5536346
N	-5.6363095	-0.5789663	0.5536346
C	7.0964194	0.6844834	0.4698743
H	7.3588745	1.7430942	0.4184650
H	7.5396856	0.2055690	1.3478659
H	7.4592548	0.1706994	-0.4297744
C	-7.0964194	-0.6844834	0.4698743
H	-7.4592548	-0.1706994	-0.4297744
H	-7.3588745	-1.7430942	0.4184650
H	-7.5396856	-0.2055690	1.3478659
O	4.4201922	-1.1166943	-1.9064097
O	-4.4201922	1.1166943	-1.9064097
C	3.0617391	-1.4264349	-2.2393509
H	2.5515628	-0.4685830	-2.3983769
H	2.9867823	-2.0215049	-3.1646212
C	-3.0617391	1.4264349	-2.2393509
H	-2.5515628	0.4685830	-2.3983769
H	-2.9867823	2.0215049	-3.1646212
H	4.8654986	-1.9386445	-1.6473611

H	-4.8654986	1.9386445	-1.6473611
H	-2.5427220	1.9637840	-1.4283715
H	2.5427220	-1.9637840	-1.4283715
Br	-0.9846086	3.7048797	2.2150561
Br	0.1534854	3.7829830	-0.9974119
Br	0.9846086	-3.7048797	2.2150561
Br	-0.1534854	-3.7829830	-0.9974119

14

C	-2.4613674	-2.6071359	0.0025196
C	-1.0732496	-2.4454841	0.1391965
C	-0.5149311	-1.3120206	0.7555369
C	-1.3987668	-0.2048522	0.9653511
C	-2.8033799	-0.4083756	0.9543891
C	-3.3255931	-1.6306902	0.4766913
C	0.8751732	-1.1130880	1.1561760
C	-0.8751732	1.1130880	1.1561760
C	0.5149311	1.3120206	0.7555369
C	1.3987668	0.2048522	0.9653511
C	1.0732496	2.4454841	0.1391965
C	-1.7437288	2.0581727	1.7343800
C	-3.1221887	1.8137730	1.8570169
C	-3.6594226	0.6245462	1.3936757
H	-3.7870323	2.5672997	2.2749690
H	-2.8770330	-3.4993905	-0.4622494
C	1.7437288	-2.0581727	1.7343800
C	3.1221887	-1.8137730	1.8570169
C	3.6594226	-0.6245462	1.3936757
C	2.8033799	0.4083756	0.9543891
H	3.7870323	-2.5672997	2.2749690
C	2.4613674	2.6071359	0.0025196
H	2.8770330	3.4993905	-0.4622494
C	3.3255931	1.6306902	0.4766913
C	4.7932412	1.8439630	0.3947547
C	5.1276513	-0.4270980	1.3917793
C	-4.7932412	-1.8439630	0.3947547
C	-5.1276513	0.4270980	1.3917793
O	5.2796231	2.8747131	-0.0592998
O	5.9155765	-1.2647679	1.8156582
O	-5.2796231	-2.8747131	-0.0592998
O	-5.9155765	1.2647679	1.8156582
N	5.5992188	0.7920699	0.8689541
N	-5.5992188	-0.7920699	0.8689541
C	7.0575363	0.9529917	0.8348434
H	7.2799457	1.9230297	0.3858036
H	7.4588372	0.8969587	1.8534384
H	7.5012556	0.1413983	0.2472283
C	-7.0575363	-0.9529917	0.8348434
H	-7.5012556	-0.1413983	0.2472283
H	-7.2799457	-1.9230297	0.3858036
H	-7.4588372	-0.8969587	1.8534384
O	3.2545977	-1.9952050	-1.3235728
O	-3.2545977	1.9952050	-1.3235728
C	2.7687226	-1.5360983	-2.5925909
H	3.6412138	-1.0961858	-3.0952194
H	2.4288545	-2.3875519	-3.2106874
C	-2.7687226	1.5360983	-2.5925909
H	-3.6412138	1.0961858	-3.0952194
H	-2.4288545	2.3875519	-3.2106874
H	2.5207448	-2.4527712	-0.8773568
H	-2.5207448	2.4527712	-0.8773568
C	1.6535931	-0.5090267	-2.4496661
H	1.9877259	0.3366173	-1.8337848

H	1.3370021	-0.1257774	-3.4294907
C	-1.6535931	0.5090267	-2.4496661
H	-1.9877259	-0.3366173	-1.8337848
H	-1.3370021	0.1257774	-3.4294907
H	-0.7755438	0.9575625	-1.9643329
H	0.7755438	-0.9575625	-1.9643329
Br	-1.1009040	3.6679362	2.5257010
Br	-0.0131478	3.7552621	-0.7298116
Br	0.0131478	-3.7552621	-0.7298116
Br	1.1009040	-3.6679362	2.5257010

15

C	-2.5856444	-2.5628064	0.4220171
C	-1.1895621	-2.4493336	0.5213405
C	-0.5732995	-1.3019969	1.0618425
C	-1.4105723	-0.1499270	1.2377200
C	-2.8248499	-0.2941107	1.2184158
C	-3.4025684	-1.5201899	0.8239321
C	0.8316116	-1.1496377	1.4239143
C	-0.8316116	1.1496377	1.4239143
C	0.5732995	1.3019969	1.0618425
C	1.4105723	0.1499270	1.2377200
C	1.1895621	2.4493336	0.5213405
C	-1.6762777	2.1401366	1.9631674
C	-3.0704460	1.9799628	2.0010989
C	-3.6479195	0.7998759	1.5625267
H	-3.7127938	2.7795352	2.3663208
H	-3.0432476	-3.4656503	0.0218237
C	1.6762777	-2.1401366	1.9631674
C	3.0704460	-1.9799628	2.0010989
C	3.6479195	-0.7998759	1.5625267
C	2.8248499	0.2941107	1.2184158
H	3.7127938	-2.7795352	2.3663208
C	2.5856444	2.5628064	0.4220171
H	3.0432476	3.4656503	0.0218237
C	3.4025684	1.5201899	0.8239321
C	4.8766138	1.6709280	0.7344009
C	5.1261111	-0.6805826	1.5073878
C	-4.8766138	-1.6709280	0.7344009
C	-5.1261111	0.6805826	1.5073878
O	5.4053138	2.6953716	0.3143689
O	5.8705826	-1.6257186	1.7517695
O	-5.4053138	-2.6953716	0.3143689
O	-5.8705826	1.6257186	1.7517695
N	5.6445281	0.5761069	1.1627701
N	-5.6445281	-0.5761069	1.1627701
C	7.1032544	0.6861270	1.0666583
H	7.3736073	1.7426518	1.1247491
H	7.5541671	0.1118787	1.8804943
H	7.4463275	0.2740802	0.1086698
C	-7.1032544	-0.6861270	1.0666583
H	-7.4463275	-0.2740802	0.1086698
H	-7.3736073	-1.7426518	1.1247491
H	-7.5541671	-0.1118787	1.8804943
O	4.4388383	-0.4855756	-1.5059150
O	-4.4388383	0.4855756	-1.5059150
C	3.6911497	0.0378593	-2.6171012
H	4.1037151	1.0409656	-2.7903258
H	3.8844171	-0.5646556	-3.5241685
C	-3.6911497	-0.0378593	-2.6171012
H	-4.1037151	-1.0409656	-2.7903258
H	-3.8844171	0.5646556	-3.5241685
H	4.2017181	-1.4193725	-1.3929874

H	-4.2017181	1.4193725	-1.3929874
C	2.1861720	0.1206358	-2.3547802
H	2.0083697	0.8202481	-1.5261201
H	1.7093167	0.5638697	-3.2437911
C	-2.1861720	-0.1206358	-2.3547802
H	-2.0083697	-0.8202481	-1.5261201
H	-1.7093167	-0.5638697	-3.2437911
C	1.5511001	-1.2314303	-2.0349674
H	1.9817736	-1.6730988	-1.1252548
H	1.6856584	-1.9499623	-2.8566983
C	-1.5511001	1.2314303	-2.0349674
H	-1.9817736	1.6730988	-1.1252548
H	-1.6856584	1.9499623	-2.8566983
H	0.4731187	-1.1378949	-1.8507789
H	-0.4731187	1.1378949	-1.8507789
Br	0.2038348	3.8585892	-0.3025601
Br	-0.9952136	3.6839630	2.8500605
Br	-0.2038348	-3.8585892	-0.3025601
Br	0.9952136	-3.6839630	2.8500605

16

C	-2.5924623	-2.5493082	0.6205423
C	-1.1956609	-2.4441603	0.7230079
C	-0.5747795	-1.2997920	1.2619351
C	-1.4090731	-0.1469558	1.4486754
C	-2.8225633	-0.2969164	1.4675527
C	-3.4044001	-1.5158212	1.0572975
C	0.8283178	-1.1507864	1.6370142
C	-0.8283178	1.1507864	1.6370142
C	0.5747795	1.2997920	1.2619351
C	1.4090731	0.1469558	1.4486754
C	1.1956609	2.4441603	0.7230079
C	-1.6604400	2.1279953	2.2181041
C	-3.0510863	1.9552728	2.3110269
C	-3.6384795	0.7807789	1.8729191
H	-3.6826882	2.7407653	2.7223349
H	-3.0546764	-3.4472874	0.2146287
C	1.6604400	-2.1279953	2.2181041
C	3.0510863	-1.9552728	2.3110269
C	3.6384795	-0.7807789	1.8729191
C	2.8225633	0.2969164	1.4675527
H	3.6826882	-2.7407653	2.7223349
C	2.5924623	2.5493082	0.6205423
H	3.0546764	3.4472874	0.2146287
C	3.4044001	1.5158212	1.0572975
C	4.8800508	1.6676699	1.0084471
C	5.1160489	-0.6446671	1.8900189
C	-4.8800508	-1.6676699	1.0084471
C	-5.1160489	0.6446671	1.8900189
O	5.4208892	2.6645711	0.5372045
O	5.8563959	-1.5711469	2.2037376
O	-5.4208892	-2.6645711	0.5372045
O	-5.8563959	1.5711469	2.2037376
N	5.6360056	0.6110475	1.5357940
N	-5.6360056	-0.6110475	1.5357940
C	7.0952020	0.7366120	1.4822185
H	7.3582453	1.7910536	1.5936124
H	7.5273542	0.1294787	2.2815656
H	7.4643594	0.3719923	0.5144534
C	-7.0952020	-0.7366120	1.4822185
H	-7.4643594	-0.3719923	0.5144534
H	-7.3582453	-1.7910536	1.5936124
H	-7.5273542	-0.1294787	2.2815656

O	4.7570027	-0.6541176	-1.0316993
O	-4.7570027	0.6541176	-1.0316993
C	4.1378912	0.0975402	-2.0893839
H	4.3951421	1.1453229	-1.8817571
H	4.5930479	-0.1707824	-3.0614773
C	-4.1378912	-0.0975402	-2.0893839
H	-4.3951421	-1.1453229	-1.8817571
H	-4.5930479	0.1707824	-3.0614773
H	4.6109739	-1.5980099	-1.1997046
H	-4.6109739	1.5980099	-1.1997046
C	2.6224463	-0.0789687	-2.1627031
H	2.1590619	0.3190946	-1.2468431
H	2.2513152	0.5438764	-2.9936192
C	-2.6224463	0.0789687	-2.1627031
H	-2.1590619	-0.3190946	-1.2468431
H	-2.2513152	-0.5438764	-2.9936192
C	2.1815865	-1.5319493	-2.3718977
H	2.4088504	-2.1209230	-1.4671743
H	2.7686647	-1.9765362	-3.1940930
C	-2.1815865	1.5319493	-2.3718977
H	-2.4088504	2.1209230	-1.4671743
H	-2.7686647	1.9765362	-3.1940930
C	0.6904172	-1.6597582	-2.6829390
H	0.3935098	-2.7056916	-2.8278164
H	0.0809197	-1.2554613	-1.8628888
H	0.4302254	-1.1019028	-3.5929585
C	-0.6904172	1.6597582	-2.6829390
H	-0.3935098	2.7056916	-2.8278164
H	-0.0809197	1.2554613	-1.8628888
H	-0.4302254	1.1019028	-3.5929585
Br	-0.9584417	3.6595079	3.1106108
Br	0.2071853	3.8815569	-0.0457463
Br	-0.2071853	-3.8815569	-0.0457463
Br	0.9584417	-3.6595079	3.1106108

17

C	-2.6015275	2.7179120	0.7185807
C	-2.5492982	1.3553056	0.7355404
C	-1.2790374	0.6411267	0.7383489
C	-0.0720929	1.4046862	0.7345230
C	-0.1459529	2.8354289	0.7160282
C	-1.3999233	3.4849883	0.7032586
C	-1.2058407	-0.7680714	0.7392347
C	1.2058407	0.7680714	0.7392347
C	1.2790374	-0.6411267	0.7383489
C	0.0720929	-1.4046862	0.7345230
C	2.5492982	-1.3553056	0.7355404
C	2.3983410	1.6087338	0.7449982
C	2.3104947	2.9693210	0.7237414
C	1.0370778	3.6083455	0.6932074
C	-2.3983410	-1.6087338	0.7449982
C	-2.3104947	-2.9693210	0.7237414
C	-1.0370778	-3.6083455	0.6932074
C	0.1459529	-2.8354289	0.7160282
C	2.6015275	-2.7179120	0.7185807
C	1.3999233	-3.4849883	0.7032586
C	1.4973042	-4.9498548	0.6632362
C	-0.9796161	-5.0675437	0.5786093
C	-1.4973042	4.9498548	0.6632362
C	0.9796161	5.0675437	0.5786093
O	2.5852596	-5.5361176	0.6549403
O	-1.9966841	-5.7590979	0.4217675
O	-2.5852596	5.5361176	0.6549403

O	1.9966841	5.7590979	0.4217675
N	0.2838730	-5.6605566	0.6253998
N	-0.2838730	5.6605566	0.6253998
C	0.3278820	-7.1213689	0.5137041
C	-0.3278820	7.1213689	0.5137041
O	-1.0947682	-4.1709067	-2.1813965
O	1.0947682	4.1709067	-2.1813965
C	-1.3140684	-2.8483035	-2.6744845
C	1.3140684	2.8483035	-2.6744845
H	-3.5488123	3.2558742	0.7125043
H	-3.4804456	0.7935589	0.7454473
H	3.4804456	-0.7935589	0.7454473
H	3.3804695	1.1431724	0.7697685
H	3.1987471	3.6006839	0.7205985
H	-3.3804695	-1.1431724	0.7697685
H	-3.1987471	-3.6006839	0.7205985
H	3.5488123	-3.2558742	0.7125043
H	1.3500502	-7.4451581	0.7240857
H	-0.3817657	-7.5595691	1.2241499
H	0.0357368	-7.4269735	-0.4996509
H	-1.3500502	7.4451581	0.7240857
H	0.3817657	7.5595691	1.2241499
H	-0.0357368	7.4269735	-0.4996509
H	-1.9273756	-4.5158790	-1.8109539
H	1.9273756	4.5158790	-1.8109539
H	-0.3561397	-2.5012963	-3.0809061
H	-2.0683231	-2.8241118	-3.4804588
H	0.3561397	2.5012963	-3.0809061
H	2.0683231	2.8241118	-3.4804588
H	1.6290755	2.1545804	-1.8738221
H	-1.6290755	-2.1545804	-1.8738221

18

C	-2.5863206	2.7332382	1.1087767
C	-2.5421213	1.3707664	1.1308232
C	-1.2750784	0.6480407	1.1256022
C	-0.0636458	1.4037723	1.1158246
C	-0.1292299	2.8344316	1.0834137
C	-1.3796590	3.4922423	1.0754335
C	-1.2109990	-0.7608947	1.1198943
C	1.2109990	0.7608947	1.1198943
C	1.2750784	-0.6480407	1.1256022
C	0.0636458	-1.4037723	1.1158246
C	2.5421213	-1.3707664	1.1308232
C	2.4081015	1.5940854	1.1059946
C	2.3282132	2.9535714	1.0540841
C	1.0579317	3.5989970	1.0308148
C	-2.4081015	-1.5940854	1.1059946
C	-2.3282132	-2.9535714	1.0540841
C	-1.0579317	-3.5989970	1.0308148
C	0.1292299	-2.8344316	1.0834137
C	2.5863206	-2.7332382	1.1087767
C	1.3796590	-3.4922423	1.0754335
C	1.4670945	-4.9572406	1.0177901
C	-1.0043708	-5.0553922	0.8885132
C	-1.4670945	4.9572406	1.0177901
C	1.0043708	5.0553922	0.8885132
O	2.5520008	-5.5491247	1.0120717
O	-2.0236200	-5.7355465	0.6894120
O	-2.5520008	5.5491247	1.0120717
O	2.0236200	5.7355465	0.6894120
N	0.2515728	-5.6589641	0.9585900
N	-0.2515728	5.6589641	0.9585900

C	0.2939671	-7.1126590	0.7779391
C	-0.2939671	7.1126590	0.7779391
O	-0.6709134	-5.0256322	-1.9697941
O	0.6709134	5.0256322	-1.9697941
C	-0.8420822	-4.0157209	-2.9729997
C	0.8420822	4.0157209	-2.9729997
C	-1.3235073	-2.6866781	-2.4009044
C	1.3235073	2.6866781	-2.4009044
H	-3.5294267	3.2770819	1.1096478
H	-3.4753162	0.8141289	1.1535618
H	3.4753162	-0.8141289	1.1535618
H	3.3871529	1.1234548	1.1280401
H	3.2184396	3.5796385	1.0221907
H	-3.3871529	-1.1234548	1.1280401
H	-3.2184396	-3.5796385	1.0221907
H	3.5294267	-3.2770819	1.1096478
H	1.2885945	-7.4616308	1.0626242
H	-0.4826735	-7.5740186	1.3952818
H	0.0987015	-7.3555942	-0.2746231
H	-1.2885945	7.4616308	1.0626242
H	0.4826735	7.5740186	1.3952818
H	-0.0987015	7.3555942	-0.2746231
H	-1.5394852	-5.2425414	-1.5887742
H	1.5394852	5.2425414	-1.5887742
H	0.1486693	-3.8979762	-3.4341085
H	-1.5295240	-4.3665139	-3.7644193
H	-0.1486693	3.8979762	-3.4341085
H	1.5295240	4.3665139	-3.7644193
H	-0.6229336	-2.3158547	-1.6411644
H	-1.4113093	-1.9297830	-3.1938627
H	0.6229336	2.3158547	-1.6411644
H	1.4113093	1.9297830	-3.1938627
H	2.3088844	2.7945299	-1.9256737
H	-2.3088844	-2.7945299	-1.9256737

19

C	-2.5152419	2.7949359	1.2150775
C	-2.5060002	1.4318579	1.2532137
C	-1.2577150	0.6792369	1.2967302
C	-0.0281014	1.4053346	1.3035013
C	-0.0578650	2.8367632	1.2685202
C	-1.2906744	3.5243568	1.2224215
C	-1.2292184	-0.7303287	1.3126458
C	1.2292184	0.7303287	1.3126458
C	1.2577150	-0.6792369	1.2967302
C	0.0281014	-1.4053346	1.3035013
C	2.5060002	-1.4318579	1.2532137
C	2.4473169	1.5333662	1.3201283
C	2.4017906	2.8954182	1.2773668
C	1.1477808	3.5725498	1.2354820
C	-2.4473169	-1.5333662	1.3201283
C	-2.4017906	-2.8954182	1.2773668
C	-1.1477808	-3.5725498	1.2354820
C	0.0578650	-2.8367632	1.2685202
C	2.5152419	-2.7949359	1.2150775
C	1.2906744	-3.5243568	1.2224215
C	1.3415620	-4.9900054	1.1458439
C	-1.1340878	-5.0341320	1.1156340
C	-1.3415620	4.9900054	1.1458439
C	1.1340878	5.0341320	1.1156340
O	2.4122484	-5.6041702	1.0733317
O	-2.1729847	-5.6923524	0.9717786
O	-2.4122484	5.6041702	1.0733317

O	2.1729847	5.6923524	0.9717786
N	0.1125468	-5.6650683	1.1580087
N	-0.1125468	5.6650683	1.1580087
C	0.1194223	-7.1219167	1.0082298
C	-0.1194223	7.1219167	1.0082298
O	-0.2303885	-4.5276300	-1.6689049
O	0.2303885	4.5276300	-1.6689049
C	0.4024461	-3.6645061	-2.6273134
C	-0.4024461	3.6645061	-2.6273134
C	0.3713088	-2.1878553	-2.2285371
C	-0.3713088	2.1878553	-2.2285371
C	-1.0466689	-1.6306631	-2.0993806
C	1.0466689	1.6306631	-2.0993806
H	-3.4439764	3.3614879	1.1724466
H	-3.4524087	0.8977809	1.2425875
H	3.4524087	-0.8977809	1.2425875
H	3.4137726	1.0383371	1.3595204
H	3.3073103	3.5001286	1.2686776
H	-3.4137726	-1.0383371	1.3595204
H	-3.3073103	-3.5001286	1.2686776
H	3.4439764	-3.3614879	1.1724466
H	1.1062096	-7.4898879	1.2969055
H	-0.6670958	-7.5491646	1.6372329
H	-0.0839629	-7.3874623	-0.0374041
H	-1.1062096	7.4898879	1.2969055
H	0.6670958	7.5491646	1.6372329
H	0.0839629	7.3874623	-0.0374041
H	-1.1914135	-4.4130541	-1.7326351
H	1.1914135	4.4130541	-1.7326351
H	1.4406351	-4.0180683	-2.6906132
H	-0.0561015	-3.7966702	-3.6250694
H	-1.4406351	4.0180683	-2.6906132
H	0.0561015	3.7966702	-3.6250694
H	0.9125023	-2.0677979	-1.2785984
H	0.9355244	-1.6194628	-2.9854238
H	-0.9125023	2.0677979	-1.2785984
H	-0.9355244	1.6194628	-2.9854238
H	-1.6245420	-2.1862070	-1.3483029
H	-1.5869370	-1.6868811	-3.0560378
H	1.6245420	2.1862070	-1.3483029
H	1.5869370	1.6868811	-3.0560378
H	1.0409976	0.5817808	-1.7770639
H	-1.0409976	-0.5817808	-1.7770639

20

C	-2.5212661	2.7913942	1.4104533
C	-2.5088894	1.4279299	1.4134852
C	-1.2590939	0.6772146	1.4472592
C	-0.0309414	1.4050036	1.4572838
C	-0.0636481	2.8370227	1.4539306
C	-1.2982611	3.5230448	1.4403457
C	-1.2279588	-0.7326903	1.4566502
C	1.2279588	0.7326903	1.4566502
C	1.2590939	-0.6772146	1.4472592
C	0.0309414	-1.4050036	1.4572838
C	2.5088894	-1.4279299	1.4134852
C	2.4441876	1.5378145	1.4494152
C	2.3954137	2.8999960	1.4324707
C	1.1398637	3.5759734	1.4325974
C	-2.4441876	-1.5378145	1.4494152
C	-2.3954137	-2.8999960	1.4324707
C	-1.1398637	-3.5759734	1.4325974
C	0.0636481	-2.8370227	1.4539306

C	2.5212661	-2.7913942	1.4104533
C	1.2982611	-3.5230448	1.4403457
C	1.3504777	-4.9905914	1.4363916
C	-1.1210010	-5.0405852	1.3598068
C	-1.3504777	4.9905914	1.4363916
C	1.1210010	5.0405852	1.3598068
O	2.4210011	-5.6078700	1.4007153
O	-2.1548644	-5.7019933	1.1920793
O	-2.4210011	5.6078700	1.4007153
O	2.1548644	5.7019933	1.1920793
N	0.1229610	-5.6667665	1.4765977
N	-0.1229610	5.6667665	1.4765977
C	0.1452247	-7.1273352	1.3740653
C	-0.1452247	7.1273352	1.3740653
O	-0.1928086	-4.9630931	-1.3819734
O	0.1928086	4.9630931	-1.3819734
C	0.3302450	-4.1266581	-2.4252452
C	-0.3302450	4.1266581	-2.4252452
C	0.0598751	-2.6383379	-2.2059654
C	-0.0598751	2.6383379	-2.2059654
C	-1.4307521	-2.2911012	-2.1514135
C	1.4307521	2.2911012	-2.1514135
C	-1.6919930	-0.7856391	-2.0740599
C	1.6919930	0.7856391	-2.0740599
H	-3.4512313	3.3570075	1.3847673
H	-3.4538179	0.8918738	1.3876234
H	3.4538179	-0.8918738	1.3876234
H	3.4119720	1.0441235	1.4551120
H	3.2996351	3.5063237	1.4145373
H	-3.4119720	-1.0441235	1.4551120
H	-3.2996351	-3.5063237	1.4145373
H	3.4512313	-3.3570075	1.3847673
H	1.0841496	-7.4886023	1.7993948
H	-0.7194138	-7.5319520	1.9068290
H	0.0850466	-7.4229290	0.3180287
H	-1.0841496	7.4886023	1.7993948
H	0.7194138	7.5319520	1.9068290
H	-0.0850466	7.4229290	0.3180287
H	-1.1611557	-4.9736593	-1.4434118
H	1.1611557	4.9736593	-1.4434118
H	1.4114316	-4.3205714	-2.4332777
H	-0.0708112	-4.4413370	-3.4068839
H	-1.4114316	4.3205714	-2.4332777
H	0.0708112	4.4413370	-3.4068839
H	0.5513246	-2.3117986	-1.2759349
H	0.5422063	-2.0811621	-3.0264231
H	-0.5513246	2.3117986	-1.2759349
H	-0.5422063	2.0811621	-3.0264231
H	-1.8846822	-2.7798117	-1.2752491
H	-1.9351053	-2.7090095	-3.0396279
H	1.8846822	2.7798117	-1.2752491
H	1.9351053	2.7090095	-3.0396279
H	-2.7622208	-0.5630492	-1.9651688
H	-1.1710540	-0.3376172	-1.2177751
H	-1.3309361	-0.2747212	-2.9774302
H	2.7622208	0.5630492	-1.9651688
H	1.1710540	0.3376172	-1.2177751
H	1.3309361	0.2747212	-2.9774302
